# Recovery of 400 Chemicals with Three Extraction Methods for Low Volumes of Human Plasma Quantified by Instrumental Analysis and In Vitro Bioassays

**DOI:** 10.1021/acs.est.3c05962

**Published:** 2023-11-21

**Authors:** Georg Braun, Martin Krauss, Beate I. Escher

**Affiliations:** † Department of Cell Toxicology, Helmholtz Centre for Environmental Research − UFZ, Leipzig 04318, Germany; ‡ Department of Effect-Directed Analysis, Helmholtz Centre for Environmental Research − UFZ, Leipzig 04318, Germany; § Environmental Toxicology, Department of Geosciences, Eberhard Karls University Tübingen, Tübingen 72076, Germany

**Keywords:** biomonitoring, exposome, low-volume, human plasma, passive equilibrium sampling, solid phase extraction, solvent precipitation

## Abstract

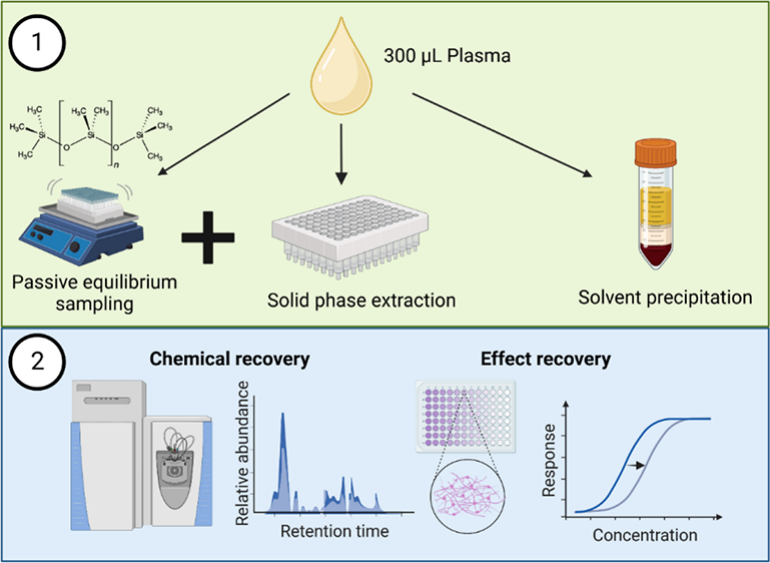

Human biomonitoring studies are important for understanding adverse health outcomes caused by exposure to chemicals. Complex mixtures of chemicals detected in blood − the blood exposome − may serve as proxies for systemic exposure. Ideally, several analytical methods are combined with *in vitro* bioassays to capture chemical mixtures as diverse as possible. How many and which (bio)analyses can be performed is limited by the sample volume and compatibility of extraction and (bio)analytical methods. We compared the extraction efficacy of three extraction methods using pooled human plasma spiked with >400 organic chemicals. Passive equilibrium sampling (PES) with polydimethylsiloxane (PDMS) followed by solid phase extraction (PES + SPE), SPE alone (SPE), and solvent precipitation (SolvPrec) were compared for chemical recovery in LC-HRMS and GC-HRMS as well as effect recovery in four mammalian cell lines (AhR-CALUX, SH-SY5Y, AREc32, PPARγ-BLA). The mean chemical recoveries were 38% for PES + SPE, 27% for SPE, and 61% for SolvPrec. PES + SPE enhanced the mean chemical recovery compared to SPE, especially for neutral hydrophobic chemicals. PES + SPE and SolvPrec had effect recoveries of 100–200% in all four cell lines, outperforming SPE, which had 30–100% effect recovery. Although SolvPrec has the best chemical recoveries, it does not remove matrix like inorganics or lipids, which might pose problems for some (bio)analytical methods. PES + SPE is the most promising method for sample preparation in human biomonitoring as it combines good recoveries with cleanup, enrichment, and potential for high throughput.

## Abstract 2

Humans are exposed to thousands of chemicals, but few are included in human biomonitoring studies. We identified suitable extraction methods to capture a diverse set of chemicals compatible with instrumental analysis and *in vitro* bioassays.

## Introduction

Human biomonitoring (HBM) has rapidly evolved over the last years and several projects and strategies have emerged with a focus on chemical exposure assessment in humans.^1,2^ An established study form are epidemiological cohort studies, which monitor participants over an extensive time frame, often years and decades (longitudinal HBM), or include large populations (cross-sectional HBM). The goal is to link behavior and environmental factors to chemical exposure and (adverse) health effects.^3−5^ While the largest number of HBM studies to date have focused on a smaller number of target chemicals,^4^ a few recent studies used suspect screening with broad chemical coverage and nontargeted analysis methods to identify and quantify chemicals in humans.^6,7^ As the exposome constitutes the entirety of chemical and nonchemical stressors over the lifetime,^8^ a comprehensive assessment of the human exposome is still out of reach but multiple proxies have been explored,^9^ and the number of chemicals identified as relevant for exposomics is ever increasing.^10^*In vitro* bioassays have only been recently introduced to capture complex mixtures of chemicals in HBM.^11^

While cohort studies produce and process large numbers of samples with a diversity of exposure scenarios, one of the major challenges is the limited amount of sample available for analysis. Human biomonitoring samples can consist of a variety of matrices, with common examples being urine,^12,13^ breast milk,^14,15^ blood (full blood, serum, and plasma),^16−18^ and even organs such as placenta^19^ or post-mortem tissues such as liver, brain, and adipose tissue.^20^ This limitation of sample quantity applies specifically to more invasive sample types like blood, where sample volumes are usually only a few hundred microliters per individuum. Depending on the study design and types of analyses for each sample, the volumes may be even lower. Hence, there is a need for sample preparation and extraction methods with broad chemical coverage, which are compatible with both instrumental analysis and *in vitro* bioassays and require only small volumes of blood or plasma.^11^ Recovery correction by using internal standards is not possible for bioassays and poses a challenge for analytical target lists with large numbers of chemicals. Therefore, it is vital to achieve robust and evenly distributed recoveries for hydrophobic to hydrophilic, neutral to charged, and persistent to nonpersistent chemicals.

We compared three common extraction methods, namely, passive equilibrium sampling (PES), solid phase extraction (SPE), and solvent precipitation (SolvPrec) and a two-step procedure combining PES with SPE (PES + SPE). Evaluation criteria were recovery of individual chemicals in relation to their physicochemical properties and compatibility with *in vitro* bioassay as well as applicability for low volumes of human plasma. Chemical recoveries were determined using target screening of more than 400 spiked chemicals by GC- and LC-HRMS, and the effect recovery was determined using the same mixtures in four cell-based bioassays. The reporter gene assays selected were the AhR-CALUX assay indicative of the activation of the aryl hydrocarbon receptor, AREc32 for oxidative stress response, and PPARγ-BLA for the activation of the peroxisome proliferator-activated receptor gamma.^21^ In addition, a neurotoxicity assay was applied that was based on cytotoxicity and neurite outgrowth inhibition in differentiated SH-SY5Y cells.^22^ The endpoints quantified by these assays are relevant for human health, but from the perspective of the extraction efficacy, it is also important that these assays respond to groups of chemicals with different physicochemical properties. The arylhydrocarbon receptor (AhR) and the peroxisome-proliferator activated receptor gamma(PPARγ) are nuclear receptors, which are activated predominately by rather large and hydrophobic chemicals^23^ and in the case of PPARγ organic anions.^24^ In contrast, the oxidative stress response is an adaptive stress response triggered by manifold molecular initiating events and consequently, the structural diversity of active chemicals is high but often smaller electrophilic chemicals are especially potent.^24^ In addition, we simulated the performance of the extracted mixtures for diverse bioassay conditions because these four bioassays are performed with different types of medium with different protein contents and with diverse protocols.

PES has been established for extracting chemicals from tissues and biological matrices.^25^ A defined mass of polymer is placed into the sample, and chemicals are extracted by direct diffusion into the polymer. The polymer of choice was polydimethylsiloxane (PDMS), which is able to extract a variety of hydrophobic and neutral compounds with relatively fast uptake kinetics due to high diffusion constants inside the PDMS^25^ but charged organic molecules only to a very limited degree.^26^ PES was already successfully applied to full blood.^27^ PES with PDMS is the most popular with lipid-rich tissues and has very slow uptake kinetics with lipid-poor tissues due to limited transport to the tissue/PDMS boundary unless the uptake rate is accelerated by vigorous stirring, which is easily possible for liquid matrices like blood and plasma. PES is a clean yet effective way to extract neutral and hydrophobic chemicals without coextracting unwanted matrix such as salts and proteins. A small fraction of lipid can be taken up into PDMS from lipid-rich matrices, but this is not a problem for plasma.^28^

SPE is a commonly used extraction method in both environmental and biomonitoring studies.^29,30^ An aqueous sample is passed through a solid sorbent in a SPE cartridge, which sorbs organic chemicals, while unwanted matrix such as inorganics and highly hydrophilic compounds percolates through the SPE cartridge and particles are retained above the frit covering the sorbent. The sorbed chemicals are subsequently eluted using organic solvents to yield a purified and enriched extract. There are many types of SPE sorbents, e.g., silica or copolymers, which are filled in plastic or glass cartridges and are available in various formats, among them miniaturized in 96-well plates.^31^ Most sorbents are optimized for extracting compounds with distinct physicochemical properties, but there are also modern copolymer materials that can also extract more hydrophilic and partially charged chemicals. The hydrophilic–lipophilic-balanced (HLB) sorbent based on the polymer *N*-vinylpyrrolidone-divinylbenzene is a versatile sorbent with good recovery established for extraction from water and wastewater. A 96-well plate version of HLB-SPE was previously applied in blood extraction^32^ and was selected in this study.

For SolvPrec, organic solvents are added to the plasma sample. Polar solvents that are miscible with water, such as acetonitrile or methanol, precipitate proteins that can be removed after centrifugation. The remaining liquid is reduced in volume by evaporation and then analyzed.^33^ As chemicals bound to the precipitated proteins would not be captured in this case, a nonmiscible apolar solvent, such as hexane, is used for chemical partitioning in this multiphasic system similar to liquid–liquid extractions. In lipid-rich body fluids and tissues, the hexane extraction is usually used as a lipid removal step, but in the case of plasma with its low lipid content the hexane extract, which also contains very hydrophobic chemicals, it can be measured with appropriate equipment or after further cleanup.^34,35^

## Materials and Methods

**Figure 1:**
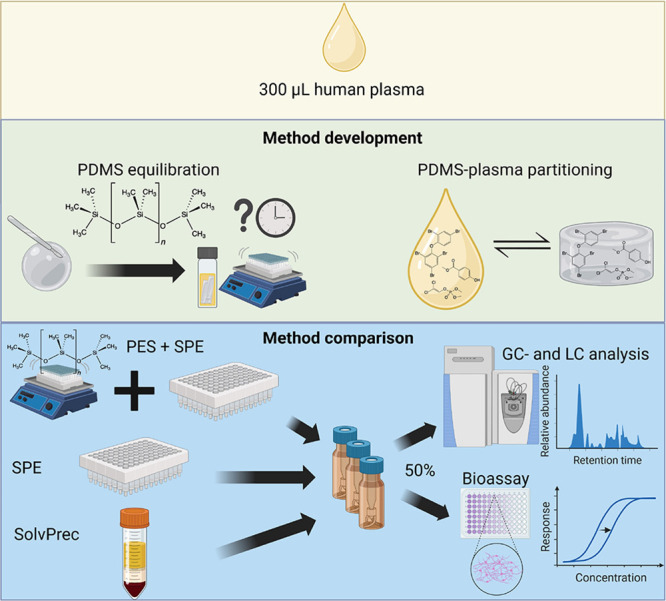
Overview of all experiments of the study. Abbreviations: passive equilibrium sampling (PES), polydimethylsiloxane (PDMS), solid phase extraction (SPE), solvent precipitation (SolvPrec), and a two-step procedure combining PES with SPE (PES + SPE).

An overview of all experiments within this study is given in Figure 1. Details regarding chemicals, materials, and devices are summarized in Supporting Information S1, Text S1. The analyte solution used for determining recoveries (spike mix) contained 1211 chemicals, each at a concentration of 500 ng/mL in 50:50 ethyl acetate (EtAc) and methanol (MeOH). All included chemicals with chemical identifiers and physicochemical properties are listed in Supporting Information S2, Table S2–1. There was an initial mix (marked in Table S2–1) that consisted of compounds mainly relevant in environmental screening. This mix was later on extended to chemicals of concern (like perfluorinated compounds) but also compounds more likely to be present in humans by being in consumer products or pharmaceuticals or prioritized for end points like neurotoxicity.^36^ The pharmaceuticals were mainly included due to their potential of artifacts in bioassays by having very specific modes of action. The mix was prepared without testing if compounds are stable, too volatile, behaved robust, or are sensitive in detection in this large composition. Procedures were not optimized for specific groups of chemicals. All methods included the evaporation of solvents and drying steps. Therefore, volatile compounds will likely not be found in the final extracts. This was considered acceptable, since the aim was to have robust methods applicable to instrumental analysis and bioanalysis. In a high-throughput bioassay environment, volatile compounds cannot be captured.^37^

The highest spiked concentrations of compounds in the samples were 40 ng/mL for LC-HRMS and 320 ng/mL for GC-HRMS, which corresponded to a maximum relative enrichment factor (REF) of 0.325 (L_plasma_/L_bioassay_) in the bioassays. A concentration of 10 ng/mL was considered to be a level of robust and detectable peaks in instrumental analysis. Thus, the selected concentration range was at the lower end by being both, detectable in instrumental analysis for a suitable range of recoveries and showing effects in bioassays. Very hydrophobic compounds, which are usually GC analytes and also prone to loss due to unspecific binding, will occur at low concentrations in real-life samples of a polar matrix like human plasma and the different concentration range between LC and GC was one way of improving detection of respective compounds while using the same extract in all test systems.

As illustrated in Figure 1, there are two main parts to this study. First, the PES method was developed and optimized by identifying the equilibration times needed to extraction a sufficient amount of chemicals as well as measuring the PDMS-plasma partitioning constants. The details of the method development experiments are accessible in Supporting Information S1, Texts S3, S6, and S7. The second and main part of the study was the comparison of the three final methods PES + SPE, SPE, and SolvPrec. These extraction methods were compared for their chemical and effect recovery.

A (phospho)lipid removal step was included in initial tests by using the Phree phospholipid and protein removal system (Phenomenex, Germany), but due to low recoveries of compounds when applying Phree cleanup (Text S2 and Figure S1–1) and the overall low lipid burden of human plasma, it was not further considered.

### SPE

The total sample volume was 300 μL with the following sample types: (A) 300 μL of phosphate buffered saline (PBS) (“blank”); (B) 300 μL of pooled plasma (“unspiked plasma”); (C) 64 μL of a 500 ng/mL compound mix, which was blown down in a nitrogen stream and then added 300 μL of pooled plasma (“spiked plasma”). All samples were prepared in glass vials.

SPE was performed with 96-well SPE plates with HLB sorbent using a negative pressure unit with a suitable manifold. 300 μL of 4% formic acid in water was added to each sample. The SPE plate was conditioned with 1 mL of ethyl acetate, 1 mL of methanol, and 1 mL of water. The sample was transferred and extracted, and the sorbent was washed with 0.5 mL of 5% MeOH in water. The plates were left under vacuum for 30 min and subsequently centrifuged at 1,500 *g* for 30 min. The plates were left overnight in a desiccator at room temperature to achieve full dryness. The plates were eluted using 1 mL of MeOH, which was collected in glass-coated 96-well plates. The eluate was evaporated to near dryness under nitrogen, transferred to 2 mL autosampler vials with 200 μL glass microinsets, evaporated to dryness, and reconstituted in 40 μL of MeOH.

### PES

A total of 500 mg of PDMS was prepared per sample by cutting 12 pieces of the size of 1 mm thickness. The total volume was 900 μL with the following sample types: (A) 900 μL of PBS (“blank”), (B) 300 μL of pooled plasma +600 μL of PBS (“unspiked plasma”), (C) 64 μL of a 500 ng/mL spike mix, which was blown down in a nitrogen stream and then added 300 μL of pooled plasma and 600 μL of PBS (“spiked plasma”). All samples were prepared in glass vials.

The diluted and spiked samples were equilibrated on an orbital shaker with 400 rpm for the respective duration at 7.5°C. After this time, the PDMS was removed, washed in Milli-Q water, and tapped dry on lint-free tissue. The PDMS was extracted for 1 day with 3.5 mL of ethyl acetate on a horizontal shaker at room temperature. This step was repeated once. The extract was evaporated to dryness under nitrogen, transferred to 2 mL autosampler vials with 200 μL glass microinsets, and reconstituted in 40 μL of MeOH. The supernatant was prepared by adding 300 μL of 4% of formic acid, and SPE was performed as described in the SPE paragraph.

### SolvPrec

The total sample volume was 300 μL with the following sample types: (A) 300 μL of PBS (“blank”), (B) 300 μL of pooled plasma (“unspiked plasma”), (C) 64 μL of a 500 ng/mL compound mix, which was blown down in a nitrogen stream and then added 300 μL of pooled plasma (“spiked plasma”). All samples were prepared in 5 mL reaction tubes.

The protocol from Pourchet^34^ that had been developed for the extraction of human milk was modified as follows: 1.8 mL of ACN were added for protein precipitation, and the samples were vortexed for 1 min. Then, 1.8 mL of hexane was added and vortexed for 1 min. The samples were centrifuged at 4000 rpm and room temperature for 15 min for phase separation. Using glass Pasteur pipettes, the lower phase (ACN and water) was removed as far as possible and transferred into glass vials. The upper phase (hexane) was also removed and placed in glass vials. Both phases were blown down by a nitrogen stream. Once the volume was low enough, the extracts were transferred into microinsets and dried under a nitrogen stream as far as possible. The water-containing phase with the remaining water was put in the desiccator overnight at room temperature to achieve full dryness. After full dryness, the samples were reconstituted in 40 μL of MeOH. ACN/water and hexane extracts were kept separate for instrumental analysis (ACN/water for LC and hexane for GC) and combined for bioassay analysis.

## Sample Analysis

### Instrumental Analysis and Recovery

The samples for LC-HRMS and GC-HRMS were prepared from 10 μL of extract. Details and further information regarding LC-HRMS and GC-HRMS method parameters can be found in the Supporting Information S1 (Text S4, Text S5, and Table S1–1 to Table S1–5). The target analytes were quantified using internal standard calibration relative to a single concentration standard (with *n* = 3). Data analysis was performed in R (version 4.1.3) using a script that read the raw files, annotated peaks via reference standards, and performed quantitative analysis using peak areas. Quality criteria were that the coefficients of variance (CV) of calculated recoveries were below 40% for replicates and below 30% for reference standards per analyte in each measurement. Due to the high number of analytes, the criteria were applied directly and individual cases were not checked for outliers. Since criteria had to be met in all possible measurements and extractions have not been tested and optimized for suitability of each analyte, this was lowering the number of compared analytes to 285–430.

Chemical recoveries per analyte *i* were calculated by using eq 1, where the peak areas found in solvent blanks (peak area_*i,*blank_) were only subtracted from the peak areas of the spiked plasma extracts (peak area_*i*,spiked plasma_) if found in >30% of all solvent blanks. The peak area_*i*,unspiked plasma+spike mix_ is the matrix-matched spike mix, which means that 16 μL spike mix (25% of the initial spike) was dried under a nitrogen stream and prepared like samples using unspiked plasma extracts. This process corrected for quenching or enhancing effects in the mass spectrometer as well as occurrence of chemicals in unspiked plasma or loss due to blow-down.
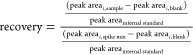
1

The chemical recovery can be further simplified to eq 2, where response ratio is the area ratio of the analyte and respective internal standard.
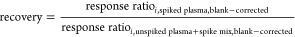
2

Recoveries were compared by using paired Friedman tests with post hoc Dunn’s tests for multiple comparisons. Further, the quantiles of log*K*_ow_ of all analytes included in the spike mix were used to bin log*K*_ow_ into four groups (*x* ≤ 1.5, 1.5 < *x* ≤ 3.01, 3.01 < *x* ≤ 4.36, *x* > 4.36). Recoveries were then sorted in these four groups to allow visualization and comparison from the perspective of hydrophobicity. Further, the p*K*_a_ values in combination with their fraction of neutral (α_neutral_) and zwitterionic (α_zwitterionic_) species at pH 7.4 were used to sort chemicals depending on the likely state of charge. The p*K*_a_ values were calculated using ACD p*K*_a_/GALAS.^38^ The bins with respect to speciation were fully negative (α_neutral_ = 0, p*K*_a_(acid) < 7.4), partially negative (α_neutral_ = 0–1, p*K*_a_(acid) < 7.4), neutral (α_neutral_ = α_zwitterionic_ = 1), partially positive (α_neutral_ = 0–1, p*K*_a_(base) > 7.4), and fully positive (α_neutral_ = 0, p*K*_a_(base) > 7.4), and recoveries were binned similar to the log*K*_ow_ approach. Multiprotic compounds were tested for their net charge at pH 7.4 and sorted accordingly into neutral, negative, or positive.

### Bioassays and Bioassay Recovery

The protocols and quality assurance and quality controls of selected biotests are accessible in the literature for AhR-CALUX, AREc32, and PPARγ-BLA,^21^ as well as SH-SY5Y.^22^ All cell lines were plated in 384-well plates and incubated for 24 h at 37 °C and 5% CO_2_ before the extracts were dosed. A maximum of 20 μL of extract was added to conic glass vials; the methanol was blown down to dryness; and 120 μL of respective cell culture medium was added. A serial dilution (1:1) was performed on 96-well dosing plates. 10 μL of each well of the dosing plate was added in duplicate on a 384-well cell plate containing 30 μL of medium and cells using an automated liquid handling robot. The cells were exposed for 24 h at 37°C and 5% CO_2_. After 24 h, cytotoxicity was quantified via cell confluency or viability staining and the reporter gene activation or morphological changes were measured according to the detailed protocols.^21,22^ To address variability, mixture and effect recovery experiments were measured in technical duplicates and biological triplicates, where each biological replicate was run with different plasma variants (Supporting Information S1, Text S1).

In analogy to the chemical recovery (eq 1), the effect concentrations EC_F_ (with F being either 10, 20, 30, or 50% depending on the highest observable effect in spike mix and unspiked plasma or an induction ratio IR of 1.5) of the spiked plasma (EC_F,spiked plasma_) as well as unspiked plasma (EC_F,unspiked plasma_) extracts and the mix (EC_F,mix_) were used to calculate effect recoveries (ER) according to eq 3.
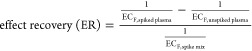
3

Before bioassay effect recovery can be calculated according to eq 3, it must be assured that the components of the mixtures acted according to the mixture model of concentration addition (CA).^39^

To test for the applicability of CA and compare with the other mixture concept of noninteracting chemicals, namely, independent action (IA), extracts of unspiked plasma were tested in binary mixtures of unspiked plasma extracts and the reference compounds narciclasine (SH-SY5Y), rosiglitazone (PPARγ-BLA), metazachlor (AREc32), and 2-amino-3-methyl-3*H*-imidazo[4,5-*f*]quinoline (AhR-CALUX).

For CA, mixture effect concentrations EC_F,CA_ can be calculated according to eq 4.
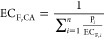
4

Here, EC_F_ depicts a benchmark effect concentration at an effect level F and p_*i*_ indicates the concentration fraction of each compound *i*.

Independent action is effect-based, and mixture effects can be calculated from the effect caused by each compound’s concentration *C*_*i*_ according to eq 5.
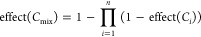
5

Since reference compounds and extracts had different units of concentration, namely, molar (M) and relative enrichment factor (REF), toxic units (TU) were used by converting EC_F,i_ to TU_i_ at a set effect level F according to eq 6. F was 10% for AhR-CALUX, PPARγ-BLA and SH-SY5Y, and an induction ratio IR of 1.5 for AREc32.
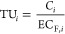
6

Mixtures were designed as equipotent on the TU scale with p_*i*_ = 0.5 at the respective effect level F. To evaluate the success of the mixture effect prediction, an index of prediction quality (IPQ) was calculated according to eq 7.^40^
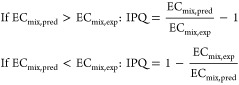
7

In eq 7, the experimental mixture effect concentrations EC_mix,exp_ were compared to the predicted mixture effect concentrations EC_mix,pred_. An IPQ of 0 would indicate a perfect prediction of the mixture effect. The IPQ and ER were calculated by resampling 9,500 times assuming normal distribution of EC_mix,pred_ and EC_mix,exp_ for IPQ and EC_F,spike mix_, EC_F,spiked plasma_ and EC_F,unspiked plasma_ for ER in the range of their respective standard errors. The equations for deriving those errors are included in the Supporting Information S1 (Text S8, eqs S1–S6).

All fits and calculations were performed in GraphPad Prism 9. For log–logistic concentration–response curves, the constraints were fixed bottom values of 0 and top values of 100. Exception was the AREc32 assay for which linear regression of the IR with a y intercept of 1. Further, only CA was used as reference mixture concept for AREc32 since CA and IA are coalescing in the linear concentration response range,^41^ and CA is the sole reference for reporter gene activation.^42^

## Results

### PES kinetics, Equilibrium and Partitioning

The distributions of recoveries after PES with PDMS for 1, 3, or 6 days of equilibration time (Table S6, Figures S1–2 in Supporting Information and Table S2–2 in Supporting Information) were very similar. This was also the case for SPE after PES (Text S6, Figure S1–3, and Table S2–3 in Supporting Information S2). The mean ranks of all combinations however were significantly different (*p* < 0.001) in the Friedman test, with exception being the distributions after 3 and 6 days of equilibration in PDMS (*p* = 0.2791). Since the mean recoveries after 3 days of equilibration time were either not significantly different (PDMS, Figure S1–2) or higher (SPE, Figure S1–3) than recoveries after 6 days of equilibration, the final equilibration time for PES in human plasma was set to 3 days. As expected, PES extracted only neutral chemicals. Extraction efficacy increased with hydrophobicity from <5% for log*K*_ow_ ≤ 1.5 to >40% at log*K*_ow_ > 4.36 (Text S6, Figure S1–2). According to theoretical considerations and previous studies with full blood,^43^ the *K*_PDMS/plasma_ should show much lower dependence on the hydrophobicity than *K*_PDMS/water_ and *K*_plasma/water_ at log*K*_ow_ > 2, which was confirmed in these experiments (Text S7).

### Comparison of PES, SPE, and the Combined Methods PES +SPE

**Figure 2:**
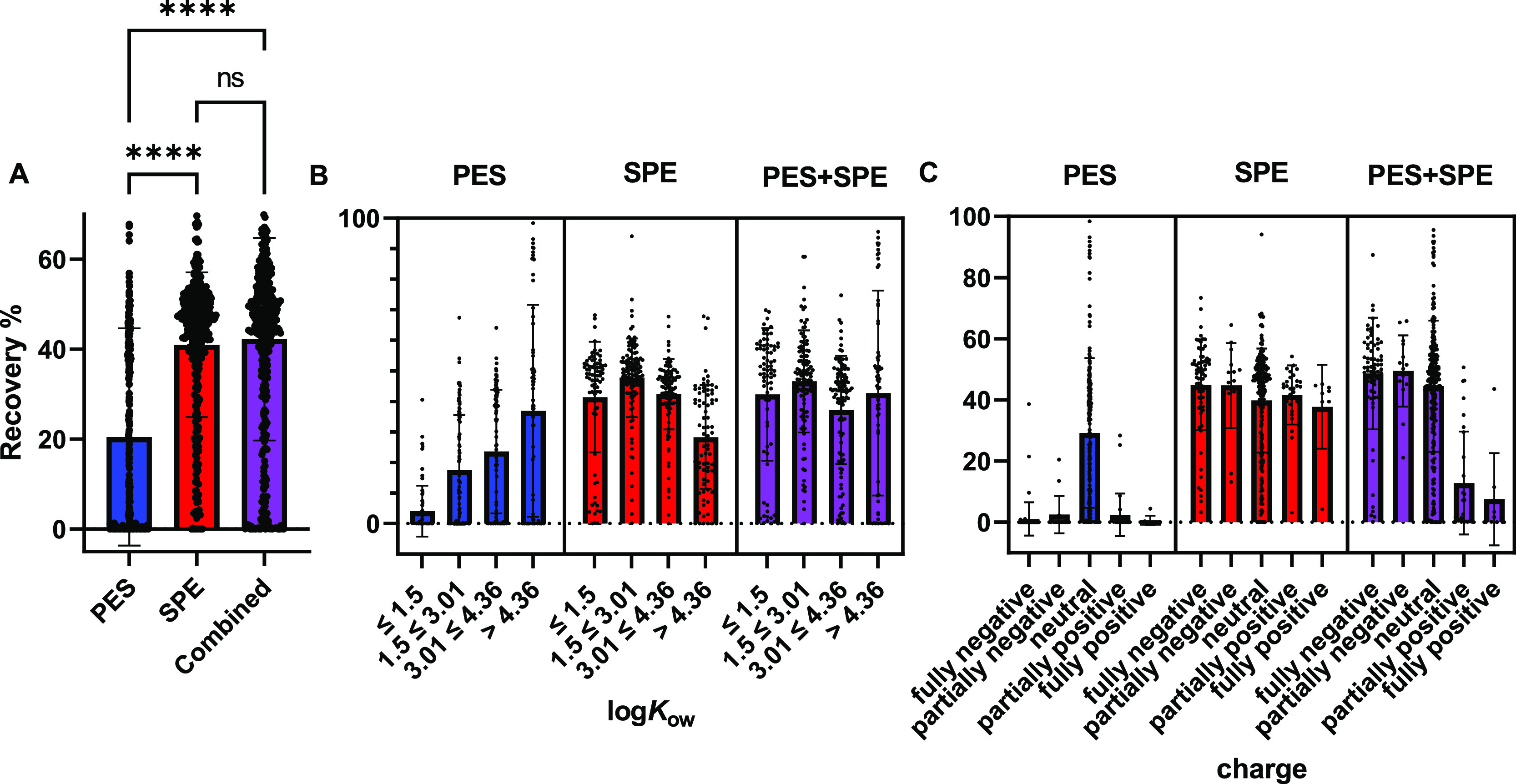
Chemical recoveries in % for PES, SPE, and PES + SPE at 3 days of equilibration. (A) Overall mean recovery and standard deviation for all analyzed compounds (*n* = 382). (B) Recoveries divided in ranges of hydrophobicity: log*K*_ow_: ≤1.5 (*n* = 77), 1.5 ≤ 3.01 (*n* = 117), 3.01 ≤ 4.36 (*n* = 111), >4.36 (*n* = 77). (C) Recoveries binned according to ionization at pH 7.4: fully negative (*n* = 68), partially negative (*n* = 15), neutral (*n* = 262), partially positive (*n* = 29), and fully positive (*n* = 8). Plotted are all individual data points as black circles, the means as boxes as well as standard deviations of the mean as error bars. log*K*_ow_ = logarithmic octanol – water partition constant. Significance tested by the paired Friedman test and Dunn’s multiple comparison. ns = not significant. Data in Table S2–44.

The recoveries at 3 days PES equilibration time (PES) and SPE after PES (Supporting Information S1, Text S6) were summed up (PES + SPE) and compared with PES-only and SPE-only, which is visualized in Figure 2 (*n* = 382) with detailed data in Supporting Information S2, Table S2–4. Considering only recoveries ≥10% PES extracted 189 compounds, SPE only extracted 353 compounds, and PES + SPE extracted 327 compounds.

The mean recoveries (± standard deviation) were 20 ± 24% for PES (including also charged chemicals that are not taken up by PES), 41 ± 16% for SPE, and 42 ± 22% for PES + SPE. The rank means were significantly different via Friedman test (*p* < 0.0001). The rank sums of PES vs SPE and PES vs PES + SPE were highly significantly different with *p* < 0.0001. The means of SPE vs PES + SPE were not significantly different with *p* = 0.3342. As demonstrated in Figure 2, due to the high diversity of chemicals, one requires both PES and SPE to cover both ends of the log*K*_ow_ range. Unexpectedly, a loss of cationic compounds was observed in PES + SPE compared to that in SPE. As PES is crucial for the recovery of neutral hydrophobic compounds, which are considered toxicologically more relevant than cationic compounds, the combined method was used in further comparisons.

### Chemical Recovery Compared between Three Final Methods the by Instrumental Analysis

**Figure 3:**
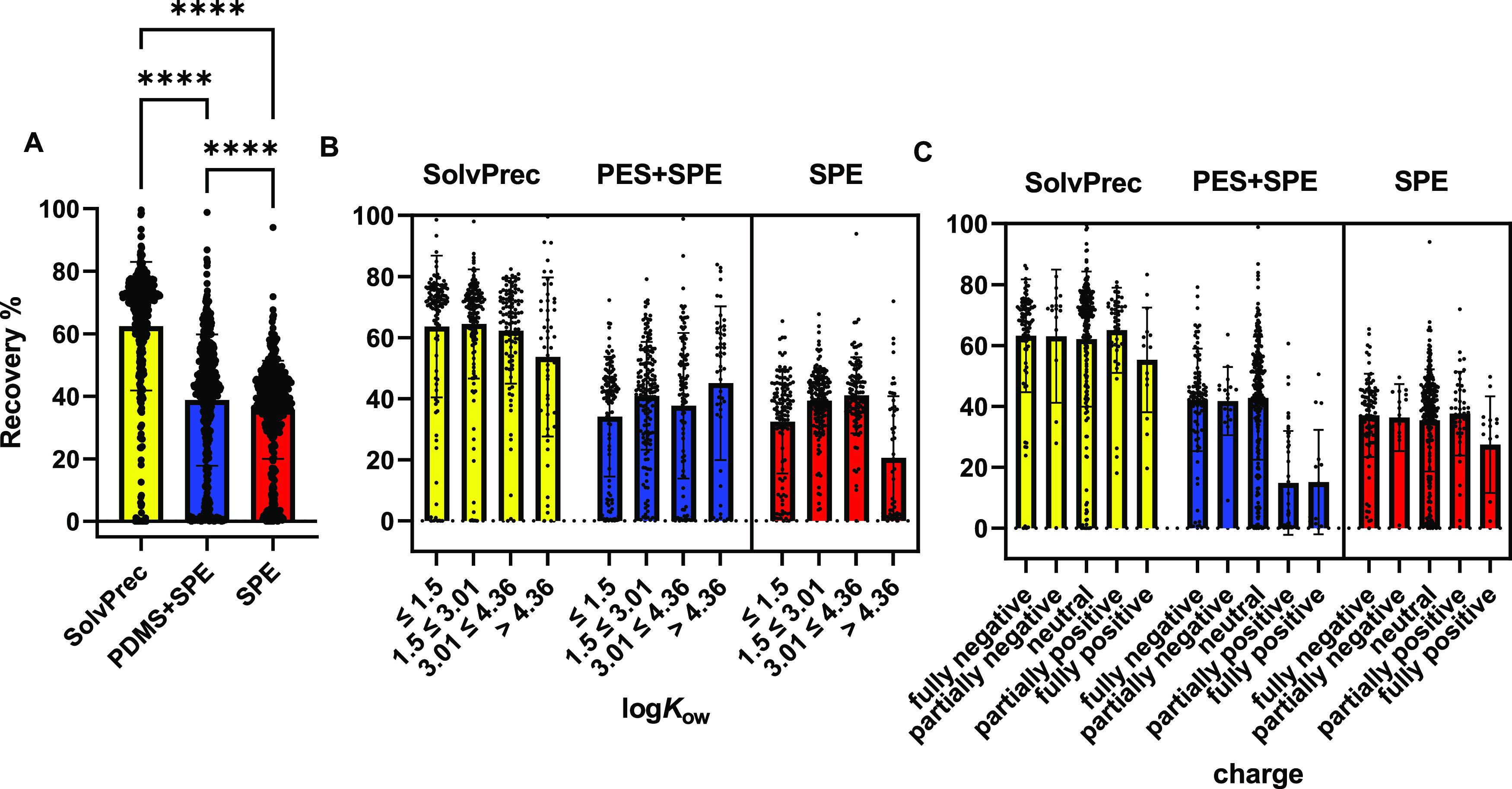
Chemical recoveries in % SolvPrec, PES + SPE, and SPE. (A) Overall mean recovery and standard deviation for all analyzed compounds per method (*n* = 430). (B) Recoveries divided in ranges of hydrophobicity: log*K*_ow_: <1.5 (*n* = 116), 1.5 ≤ 3.01 (*n* = 158), 3.01 ≤ 4.36 (*n* = 105), >4.36 (*n* = 51) per method. (C) Recoveries binned according to ionization at pH 7.4: fully negative (*n* = 89), partially negative (*n* = 17), neutral (*n* = 266), partially positive (*n* = 43), and fully positive (*n* = 15). SolvPrec = solvent precipitation, SPE = solid phase extraction, PES = passive equilibrium sampling. log*K*_ow_ = logarithmic octanol – water partition constant. Significance tested by paired Friedman test and Dunn’s multiple comparison. Data are given in Table S2–5.

Three-day PES + SPE was compared with SPE and SolvPrec. The mean chemical recoveries ± standard deviation were 62 ±21% for SolvPrec, 39 ± 21% for PES + SPE, and 35 ± 16% for SPE as visualized in Figure 3 (*n* = 427) with detailed data in Supporting Information S2, Table S2–5. Considering only recoveries ≥10% SolvPrec extracted 401 compounds, PES + SPE extracted 359 compounds, and SPE recovered 375 compounds out of 427. Paired Friedman test revealed significant difference between average group ranks with *p* < 0.0001. SolvPrec had the highest overall recovery and highly significant difference to the other methods with *p* < 0.0001. The recovery of PES + SPE was also significantly higher than SPE with *p* < 0.0001.

When recoveries were binned according to log*K*_ow_, all three methods showed rather even distributions of recoveries. The only exception was SPE, which has a tendency of lower recoveries for hydrophobic compounds with log*K*_ow_ > 4.36 (Figure 3). In terms of recovery under consideration of the ionization state, SolvPrec and SPE show even distributions, while PES + SPE showed substantial loss of positively charged chemicals.

### Mixture Experiments

The mixture experiments served to justify that effect recovery can be calculated with eq 3 because this equation is only valid for concentration-additive mixture effects. The concentration–response curves of method blanks (Figure S1–5), reference compounds (Figure S1–6), and unspiked samples (Figure S1–8 to Figure S1–11) and the binary mixtures (Figures S1–12 to Figure S1–15) for each bioassay can be accessed in the Supporting Information S1, Text S11. The respective EC_F_ and IC_F_ (for cytotoxicity) are accessible in Table S2–-6 in Supporting Information.

The comparisons of predicted and measured concentration–response curves of the binary mixture of the unspiked plasma + reference samples in Figures S1–12 to Figure S1–15 showed a good agreement between the triplicate experiments per cell line and the predictions for both CA and IA, in most cases. The index on prediction quality (Tables S1–6 and S1–7 in Supporting Information S1) scattered around ± 1 for the majority of combinations of bioassays and extraction methods. This confirms the applicability of CA for bioassays, and thus the calculation of effect recoveries for spiked serum samples with eq 3 was deemed valid.

### Effect Recovery Quantified in Cell-Based Bioassays

The concentration response curves of the spiked sample are accessible in the Supporting Information S1 and Text S11 (Figures S1-16–19) and the thereof derived effect concentrations are listed in Supporting Information S2 and Table S2–6. The effect recoveries (ER) ± standard deviation (SD) as calculated with eq 3 per bioassay and end point are summarized in Supporting Information S1, Text S10, and Table S1–8 and visualized in Figure 4.

**Figure 4:**
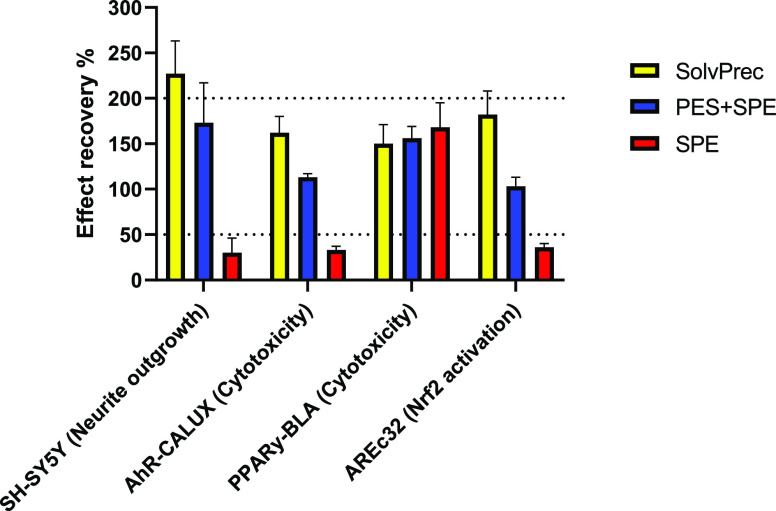
Mean effect recoveries per method and bioassay/cell line calculated with eq 3 from the effect concentrations from Table S2–6. Dotted lines indicate the acceptable interval of 50–200% effect recovery. Standard deviation is shown as error bars. Data accessible from Supporting Information S1, Text S10, Table S1–8.

Analogously to chemical recoveries, effect recoveries can be used to characterize the performance of extraction methods based on how much specific effects remain in spiked samples compared to unspiked samples and blanks. PES + SPE and SolvPrec showed mean effect recoveries ranging from 100–200% in all tested cell lines, while SPE spanned 30–175% effect recovery (Figure 4). SolvPrec and PES + SPE were for the most part within the accepted recovery range of 50–200% in all cell lines, while SPE was only high enough in the PPARγ-BLA cell line (Figure 4). This is indicative that PES + SPE and SolvPrec are extraction methods that are able to extract a significant portion of specific effects in a variety of bioassays, while SPE was not.

## Discussion

The three selected extraction methods PES + SPE, SPE, and SolvPrec were compared for their efficacy of recovering a large variety of chemicals (chemical recovery) as well as ability to extract chemicals relevant for specific effects in diverse cell-based bioassays (effect recovery). Several aspects need to be considered in interpreting these results.

### Chemical Recoveries

PES had an extraction efficacy lower than that predicted during experimental planning. Prior to the present study, *K*_PDMS/plasma,*i*_ was not known, but as detailed in Text S7, they can be predicted by a mass balance model (SI, equation S4) from *K*_PDMS/lipid_. The maximum experimental *K*_PDMS/plasma,*i*_ aligned well with the mass balance model (*K*_PDMS/plasma_ = 13.7 at log*K*_ow_ > 4.36), but many individual log*K*_PDMS/plasma,*i*_ were much lower than predicted (Figure S1–4), which explains that the mean recovery of neutral chemicals was lower than 100%. Unfortunately, the mass to volume ratio of PDMS to plasma cannot be further increased for practical reasons because the 500 mg of PDMS already requires 300 μL of plasma to be diluted with 600 μL of PBS to ensure full coverage of the PDMS by the plasma solution. The additional SPE will increase the recovery of neutral and hydrophilic chemicals, so the combination of PES + SPE still has a good extraction efficacy (Figure 2). Most importantly, the combination of PES and SPE led to the hydrophobicity-independent recovery of chemicals. This means that the concentration ratios between chemicals were not changed by the extractions, which is important for the bioassays.

As expected, SPE had the overall lowest recoveries and decreased recoveries for hydrophobic chemicals with log*K*_ow_ > 4.36 but SPE is the method best suited for charged organic chemicals (Figure 3). PES + SPE was significantly better than SPE alone but had a strong deficit in the recovery of positively charged chemicals (Figure 3). The loss of certain cationic compounds, including the pesticides spinosad (spinosyn A), emamectin B1a, mepiquat, and pharmaceuticals like metformin, sertraline, amitryptilin, or oxybutynin, was unexpected and can have multiple reasons. As SPE did not cause such a high loss, we conclude that the organic cations were lost, presumably degraded during the 1 and 3 days of PES. PES was shown to be able to extract small fractions of bulky multiprotic and also cationic compounds in the past, with assumptions of charge delocalization.^26^ The acidification step in the SPE will also likely affect recoveries of cationic compounds, but as shown for SPE, the recoveries binned according to the chemicals’ charge were rather balanced (Figure 3). The recoveries for these compounds were already low after 1 day of PES equilibration, so no optimization in terms of time was possible.

Given the unexplained reduced recovery of organic cations, one must consider which chemicals are more relevant for the toxicity end points selected. Hydrophobic chemicals are likely present at already rather low concentrations in an aqueous matrix like human plasma; therefore, high recovery is very important, especially since hydrophobic chemicals usually have a higher contribution to toxicity compared to very hydrophilic chemicals, act as baseline toxicants, and are relevant in bioaccumulation.^44−46^ Nevertheless, compounds like spinosad can be relevant for end points like oxidative stress and (developmental) neurotoxicity.^47,48^ However, if compounds are not stable for an equilibration time of 1 day at 7.5°C and pH 7–8, their relevance for human exposure assessment is also to be questioned. Focus on known parent compounds is also one limiting factor of using target screening, since potentially more toxic metabolites could have formed.

The good chemical recovery (Figure 3) of the solvent precipitation (SolvPrec) is likely due to the simplicity of the protocol where only proteins are removed from the plasma. However, this means that matrices such as salts or lipids are also not removed from the samples if both the aqueous ACN and the hexane phase are used for analysis. Only using the aqueous ACN phase would mean the removal of many hydrophobic compounds, substantially reducing the chemical diversity in the extract, with a bias toward hydrophilic chemicals, which are often of lower toxic potency. The hexane phase contains the more hydrophobic chemicals, which are toxicologically relevant but also lipids. Coextracted lipids disturb chemical analysis and bioassays.^28^ Hence, SolvPrec as used in this study can only be applied to already rather clean samples with a low lipid burden and osmolality, such as plasma. The use of special injector systems like thermal desorption units (TDU) for GC-MS or diluting or filtering samples for LC-MS and bioassays can become necessary for analysis if SolvPrec is used for more challenging sample matrices containing more unwanted background like urine, human milk, or whole blood samples. The (phospho)lipid removal step that we also evaluated (Text S2) would also be a valid step for lipid removal. However, as with any additional step, a loss of compounds was expected to occur, and the experimental findings deemed that this loss was too high (Supporting Information S1, Figure S1–1). Due to the low lipid content of plasma, any coextracted lipid did not disturb the chemical analysis or the bioassays, and therefore, lipid removal was discontinued for this type of matrix.

SolvPrec showed balanced recoveries in both physicochemical aspects, log*K*_ow_ and charge (Figure 3). Hence, if both phases are used for analysis, this extraction method in combination with the overall higher recoveries can be considered the best and unbiased choice. However, it is almost like a direct dosing or injection of plasma into bioassays and instruments after just removing the proteins by precipitation. If GC is included as part of the chemical analysis, the extract should not contain any water to avoid damage to the columns and injector systems. PES + SPE and SPE allow further enrichment via blow-down of solvent, since all water is removed during extraction. In contrast, blowing down the aqueous ACN phase in SolvPrec requires much harsher conditions to remove water, such as longer blow down times and extended drying steps.

Although the overall recoveries were lower than for SolvPrec, PES + SPE and SPE can be considered as more selective extractions since both hydrophobic matrix, such as lipids, and hydrophilic matrix, such as salts, will be reduced or even removed using these extractions methods.^27^

### Effect Recoveries

CA and IA had very similar predictions, so CA could be used as the reference case for all cell lines. The calculated IPQs in the mixture experiments scattered for most extraction methods were around 1 (Table S1–6 and Table S1–7), meaning 2-fold deviation, which is an empirical acceptable deviation in bioassays.^49^ The main reason why effect recoveries have this larger acceptable range (50% to 200%) in comparison to chemical recovery (usually 80–120%) is that concentration–response curves are mainly on a logarithmic scale yielding logEC_10_ values (Figures S1-6–19, with the exception of AREc32, which is on a linear concentration scale and yields EC_IR1.5_). A recovery of 50 to 200% is only ±0.3 log unit, which is well within the biological variability of a bioassay. Nevertheless, they provide important information about the hazard-based relevance of chemical mixtures and can help identify extraction methods as demonstrated in this study, which cover the biologically relevant contaminants. As concentration addition is valid, the effect recovery could be calculated with eq 3.

Further, the neurotoxicity assay with SH-SY5Y exhibited the highest variability of IPQ but was also the only bioassay with high-content imaging, which is a new application for extracts from complex matrices and might require further optimization, while the other assays were using reporter genes and luminescence measurements with a plate reader. The relatively high deviation between the different plasma variants for the effect recovery in SH-SY5Y could also be due to high sensitivity for (chemical) differences between the unspiked samples (Supporting Information S1, Table S1–8).

The overall declining sequence of effect recoveries of SolvPrec > PES + SPE > SPE showed a similar trend as the distributions of chemical recovery (Figure 3), which is plausible. All methods were able to extract a significant portion of bioactive components of the samples, especially SolvPrec and PES + SPE, which usually had recoveries >100% (Supporting Information S1, Table S1–8). This advantage over the SPE could be due to the improved recoveries of hydrophobic chemicals because those usually have a higher leverage in terms of the (toxic) effect because they have a much higher baseline toxicity. The use of cytotoxicity for calculating effect recovery in PPARγ-BLA and AhR-CALUX was not optimal since the specificity of the cell lines cannot be addressed, but given that the spike mix contained 1211 chemicals, the nonspecific effects of this large mixture was unfortunately masking potential specific effects, which is also not an uncommon observation in extracts from complex matrices and therefore represents a realistic scenario.

Considering the robust recoveries of toxicologically relevant contaminants, the lower likelihood of matrix effects, and the overall very convincing effect recoveries, we recommend PES + SPE as the extraction method for monitoring mixture effects of chemicals in human plasma in future cohort and biomonitoring studies.

## Supporting Information Available

Additional information regarding conditions of instrumental analysis; results of phospholipid removal steps; split results for equilibrium testing for PES and SPE; calculation of prediction errors; indices on prediction quality; concentration response curves of the bioassays; additional information about consumables, lipid-removal steps, instrumental analysis, internal standards, experiments and results to derive the PDMS equilibration time and PDMS-plasma partition constants, calculation of errors of benchmark concentrations and mixture effect modeling, index on prediction quality, concentration–response curves (PDF)
Details about spike mix composition; instrumental recovery per compound like recovery, errors, *n*, instrument and mode, reasons for exclusions, effect concentrations in the bioassays, PDMS-plasma partition constants; information about the analyzed compounds, recoveries and benchmark concentrations of the respective samples (XLSX)

## Abbreviations

ACN: acetonitrile
CA: concentration addition
EC: effect concentration
ER: effect recovery
EtAc: ethyl acetate
GC: gas chromatography
Hex: hexane
HRMS: high-resolution mass spectrometry
IA: independent action
IPQ: Index on prediction quality
LC: liquid chromatography
log*K*_ow_: logarithmic octanol–water partition constant
log*K*_PDMS/plasma_: Logarithmic PDMS-plasma partition constant
MeOH: methanol
MS: mass spectrometry
PDMS: polydimethylsiloxane
PES: passive equilibrium sampling
SD: standard deviation
SolvPrec: solvent precipitation
SPE: solid phase extraction
TDU: thermal desorption unit
