# Supplementary material for: Recovery of 400 Chemicals with Three Extraction Methods for Low Volumes of Human Plasma Quantified by Instrumental Analysis and In Vitro Bioassays
Source: Environ Sci Technol. 2023 Nov 21;57(48):19363–73. doi: 10.1021/acs.est.3c05962 (PMC10702517; doi:10.1021/acs.est.3c05962)
Supplement: Supplementary file 1 — es3c05962_si_001.pdf [file es3c05962_si_001.pdf]

Supporting Information S1

Recovery of 400 chemicals with three extraction methods for low volumes of human plasma quantified by instrumental analysis and in vitro bioassays

Georg Braun*[1], Martin Krauss[2] and Beate I. Escher[1,3]

[1]Department of Cell Toxicology, Helmholtz Centre for Environmental Research – UFZ, Leipzig, Germany

[2]Department of Effect-Directed Analysis, Helmholtz Centre for Environmental Research – UFZ, Leipzig, Germany

[3]Environmental Toxicology, Department of Geosciences, Eberhard Karls University Tübingen, Tübingen, Germany

9 Tables

19 Figures

31 Pages

Plus 9 Tables in Supplementary Information S2 (xslx file)

Table of content

Tables:

Table S1-1: Temperature gradient and split of the TDU unit
Table S1-2: Temperature gradient of the GC oven
Table S1-3: Internal standard mix used for GC-HRMS
Table S1-4: Solvent gradients for LC-HRMS analysis in positive and negative mode
Table S1-5: Internal standard mix used for LC-HRMS
Table S1-6: Index on prediction quality and standard deviation for concentration addition
Table S1-7: Index on prediction quality and standard deviation for independent action
Table S1-8: Effect recoveries (ER) per method and bioassay/cell line calculated with equation (3) from the effect concentrations in Table S2-6.

Figures:

Figure S1-1: Chemical recovery distributions after (phospho)lipid removal using Phree®.
Figure S1-2: Chemical recoveries in % for PES at different timepoints.
Figure S1-3: Chemical recoveries in % for SPE after PES for different durations (1, 3 or 6 days).
Figure S1-4: PDMS-plasma partition constants log$K_{PDMS/plasma}$ of the neutral chemicals (n = 352) as a function of the octanol-water partition constant logK_{ow}.
Figure S1-5: Concentration-response curves of method blanks in all cell lines.
Figure S1-6: Concentration-response curves of single reference chemicals used for the binary mixture experiments.
Figure S1-7: Concentration-response curves of the spike mix in all cell lines.
Figure S1-8: Concentration-response curves of unspiked plasma samples in SH-SY5Y.
Figure S1-9: Concentration-response curves of unspiked plasma samples in PPARγ-BLA.
Figure S1-10: Concentration-response curves of unspiked plasma samples in AhR-CALUX.
Figure S1-11: Concentration-response curves of unspiked plasma samples in AREc32.
Figure S1-12: Concentration-response curves of unspiked plasma + reference in SH-SY5Y.
Figure S1-13: Concentration-response curves of unspiked plasma + reference in PPARγ-BLA.
Figure S1-14: Concentration-response of unspiked plasma + reference in AhR-CALUX.
Figure S1-15: Concentration-response of unspiked plasma + reference in AREc32.
Figure S1-16: Concentration-response curves of spiked plasma samples in SH-SY5Y.
Figure S1-17: Concentration-response curves of spiked plasma samples in PPARγ-BLA.
Figure S1-18: Concentration-response curves of spiked plasma samples in AhR-CALUX.
Figure S1-19: Concentration-response curves of spiked plasma samples in AREc32.

Text S1. Chemicals and Consumables

Ethyl acetate (EtAc) and methanol (MeOH) of LC-MS grade were bought from Honeywell. LC-Grade water (water) was bought from Th. Geyer, Hamburg, Germany (418.1000). Phosphate buffered saline (PBS) was prepared by adding 8.2 g of NaCl (Roth), 0.2 g of KCl (Merck), 1.44 g of $Na_2HPO_4{\cdot}2H_20$, and 0.25 g of KH_2PO_4 (Merck) to 100 mL of water. Milli-Q water (MilliQ) was collected from a respective filter device. Acetonitrile (ACN) and hexane (Hex) were bought from (Merck)

Shell vials and caps used in passive sampling were bought from neoLab (7-0743). 15 mL Tubes were bought from Greiner (10384601). Glass vials (7663231) with inserts (7614088) from LabSolute. Caps were bought from Phenomenex (AR0-8952-13-M) and Wicom (43941/B).

Polydimethylsiloxane (PDMS) was bought from Shielding Solutions (SSP-M823-040) and was cut in disks with 125.6 ± 1.17 mg (n = 80). The disks were put in a Soxhlet device with ethyl acetate for three consecutive days to remove impurifications and stored in ethyl acetate until use.

Human pooled plasma was bought from biowest (S4180, different batches "Plasma1" and "Plasma3") and Innovative Research (IPLANAH, "Plasma2"). The PDMS-plasma partitioning constant experiments were done with "Plasma1".

SPE 96-well plates with 10 mg per well of hydrophilic-lipophilic balanced (HLB) sorbent were bought from Macherey-Nagel (738921.010M). Phree® plates were bought from Phenomenex (8E-S133-TGB).

For equilibration an orbital shaker (TiMix 2, Edmund Bühler GmbH) and a horizontal shaker (HS 501 digital, IKA-Werke) were used. The centrifuge for 96-well plates was a Multifuge X1R, Heraeus. Hamilton Star liquid handling system. Blow-down of solvents was performed on an XcelVap (Horizon) automated evaporation system.

Text S2. Lipid removal

Lipids may can cause problems in both, bioassays and instrumental analysis by causing unspecific effects,[1] binding chemicals and therefore reducing the bioavailability in bioassays,[2] or influencing ionization efficiency in the mass spectrometer.[3, 4] In this study we evaluated the Phree® phospholipid removal plates[5] in combination with SPE.

The total sample volume was 300 µL with the following sample types: A) 300 µL PBS (blank) B) 300 µL pooled plasma (unspiked) C) 64 µL of a 500 ng/mL compound mix which was blown-down, then adding 300 µL of pooled plasma (spiked). All samples were prepared in glass vials.

300 µL of sample were added to the 96-well Phree® phospholipid removal plate. 900 µL ACN with 1% FA were added and samples were shaken at 300 rpm using an orbital plate shaker for 2 minutes. The plate was placed on top of a glass coated 96-well plate and elution took place by centrifugation at 500 g for 10 minutes. ACN was blown-down and 900 µL of water were added. Then, SPE was performed without adding 4% FA as described in the Solid-Phase-Extraction paragraph.

After (phospho)lipid removal followed by SPE, the overall mean chemical recovery was 2.53 ± 5.14 % (Figure S1-1A) with no significant difference for the selected logK_{ow} intervals (Figure S1-1B).

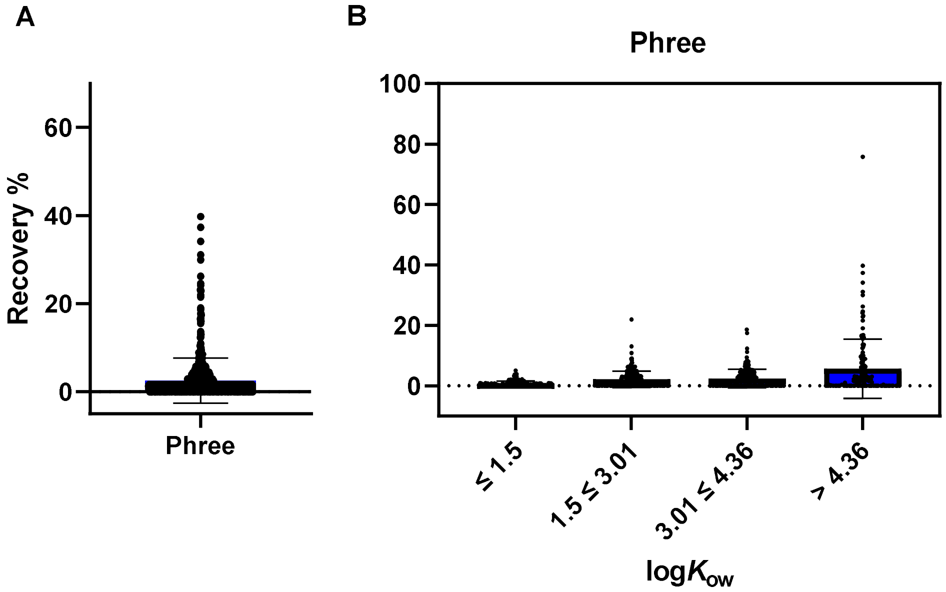

Figure S1-1: Chemical recovery distributions after (phospho)lipid removal using Phree®.
(A) Overall mean recovery and standard deviation for all analyzed compounds (n = 843). **(B)** Recoveries separated in bins of logK_{ow} ≤ 1.5 (n = 217), 1.5 ≤ 3.01 (n = 235), 3.01 ≤ 4.36 (n = 222), > 4.36 (n = 166).

Text S3. PDMS-plasma partition constants

Independent experiments were performed to derive the PDMS-plasma partitioning constants ($K_{PDMS/plasma.}$) Samples were prepared from 64 µL of a 500 ng/mL spike mix, which was blown down in a nitrogen stream and resolubilized in 300 µL of pooled human plasma. PES was added as 10 mg, 50 mg, 100 mg, and 150 mg pieces in triplicates. The samples were equilibrated in batch one for three days and batch two for eight days at 7.5°C and 400 rpm on an orbital shaker. Afterwards the PDMS was removed, washed in MilliQ water and tapped dry. The PDMS was extracted twice using ethyl acetate in the volumes of 1.05 mL (150 mg PDMS), 0.7 mL (100 mg PDMS), 0.35 mL (50 mg PDMS), and 0.07 mL (10 mg PDMS). The extract was evaporated to dryness under nitrogen, transferred into 2 mL autosampler vials with 200 µL glass micro inserts and reconstituted in 40 µL of MeOH. Samples were measured by GC-HRMS and LC-HRMS.

For the calculation of the $K_{PDMS/plasma}$ a mass balance equation for partitioning between PDMS and the plasma was set up, assuming negligible loss due to evaporation or binding to compartments besides plasma or PDMS. Since 64 µL of 500 ng/mL spike mix were used and 25% of extract were injected into the LC and GC systems, the total mass with 100% chemical recovery equals 8 ng. The respective concentrations in PDMS ($C_{PDMS,i}$) and plasma ($C_{plasma,i}$) per chemical *i* were calculated by equation S(1) and the concentration remaining in plasma with equation S(2). The factor 1.025 is the correction for density (g/mL) of human plasma.

$$C_{PDMS,i}\left(\frac{ng}{g}\right) = \frac{8\ ng \times Recovery_i}{m_{PDMS}(g)} \qquad S(1)$$

$$C_{plasma,i}\left(\frac{ng}{g}\right) = \frac{8\ ng \times (1\text{-}Recovery_i)}{V_{plasma}(mL) \times 1.025(\frac{g}{mL})} \qquad S(2)$$

The $K_{PDMS/plasma}$ of chemical i can be described as the concentration ratio (equation S(3)).[6]

$$K_{PDMS/plasma,i} = \frac{C_{PPDMS,i}}{C_{plasma,i}} \qquad S(3)$$

The final PDMS partition constant $K_{PDMS/plasma,i}$ were calculated from the mean of all experiments.

The experimental $K_{PDMS/plasma}$ were compared with theoretical predictions from a simplified mass balance model (equation S(4)) adapted from Baumer et al.,[7] where the partitioning constant between PDMS and lipids, $K_{PDMS/lipid}$, was approximately constant over a wide hydrophobicity range and amounted to 10,[8] the partition constant between lipids and serum proteins, $K_{lipid/protein}$, was approximately constant over a wide hydrophobicity range and predicted as 20.[7] The mass fraction of lipid, mf_{lipid} was measured as in Baumer et al.[7] and amounted to 0.00446, that of proteins $mf_{protein}$ was 0.05647. The remainder was assumed to be non-binding and assigned to water, mf_{lwater}, and was calculated as mf_{water} = 1- mf_{lipid} - $mf_{protein}$ = 0.93907.

$$K_{PDMS/plasma} = \frac{K_{PDMS/lipid}}{\left(mf_{lipid} + \frac{mf_{protein}}{K_{lipid/protein}} + \frac{mf_{water}}{K_{lipid/water}}\right)} \qquad S(4)$$

Text S4. GC-HRMS analysis

Samples were prepared by adding 10 µL of extract and 5 µL of 250 ng/mL internal standard mix to 10 µL of MeOH. Pure MeOH was used as solvent blanks. GC-HRMS analysis was performed on Thermo Trace 1310 GC system and Q Exactive™ Orbitrap™. A J&W DB-5ms Ultra Inert GC Column, 30 m x 0.25 mm, 0.25 µm film thickness was used for separation. With helium as carrier gas. For injection, we used a Gerstel multi-purpose autosampler with thermal desorption unit (TDU) and a Gerstel cold injection system (CIS). The injection volume was 2 µL into thermodesorption tubes equipped with glass inserts. The TDU was heated according to Table S1-1. The CIS was initially cooled to -20°C and heated up quickly to 300°C using a rate of 720°C/min in splitless mode at 75 kPa for desorption. The CIS transfer temperature was 320°C. This was the injection of the sample into the GC system which had a subsequent oven temperature gradient as shown in Table S1-2 at a constant flow of 1.2 mL/min.

The use of a TDU in combination with CIS is applied to reduce the injection of matrix by initially desorbing the sample into the CIS leaving behind non-desorbing components of the sample in the glass tubes (which can be considered as single use liners) and is the standard injection system used for this instrument.

During measurements the ion source temperature was 200°C and transfer line temperature was 250°C. Electron ionization was used at an emission current of 50 µA and an electron energy of 70 eV.

The instrument was operated in full scan MS1 mode with a m/z range from 60 - 810 and a nominal resolving power of 60,000 referenced to m/z 200.

Table S1-1: Temperature gradient and split of the TDU unit

**Final temperature (°C)**	**Rate (°C/Min)**	**Holding time (Min)**	**Purge (mL/Min)**	**Split**
80	0	4	60	TRUE
300	300	5	0	FALSE

Table S1-2: Temperature gradient of the GC oven

**Rate (°C/Min)**	**Final temperature (°C)**	**Holding time (Min)**
0	60	1
30	150	0
6	186	0
4	300	11.5

The internal standard mix consisted of the compounds listed in Table S1-3 and was at 1 µg/mL. Internal standards were assigned by nearest retention time or to the respective analyte without isotope label.

Table S1-3: Internal standard mix used for GC-HRMS

Tonalide-D3	Hexachlorobenzene-13C6
BDE99-13C12	Pyrene-D10
PCB153-13C12	Etofenprox-D5
PCB180-13C12	1,2,4-Trichlorobenzene-D3
PCB28-13C12	Decachlorobiphenyl-13C12
PCB52-13C12	2,2',4,4'-Tetrabromodiphenylether-13C12
Gamma-HCH-13C12	Pyrene-D10
Acenaphthene-D10	Naphthalene-D8
Benzo(a)pyrene-D12	PCB101-13C12
Phenanthrene-D10	PCB118-13C12
Perylene-D12	PCB138-13C12
Chrysene-D12	2,4'-DDT-D8

Text S5. LC-HRMS analysis

LC-HRMS was performed on Thermo Ultimate 3000 LC system with an electrospray ion source and a Q Exactive Plus quadrupole-Orbitrap instrument. The column was an ACQUITY UPLC® BEH C18, 100 x 2 mm, 1.7 µm particle size (Waters). Eluents were 1 mM ammonium formate with 0.1% FA in water and methanol for positive mode, and for negative mode 2 mM ammonium bicarbonate in water and 95:5 MeOH:water. 90:10 water:MeOH was used for solvent blanks. Samples were prepared by adding 10 µL of extract and 10 µL of 100 ng/mL internal standard mix to 180 µL of water. The injection volume was 50 µL. The heated ESI source and the transfer capillary were both operated at 300°C, the spray voltage was 3.8 kV (positive mode) or 3.5 kV (negative mode), the sheath gas flow rate was 45 a.u. and the auxiliary gas flow rate 1 a.u. Separate runs were conducted in positive and negative ion mode combining a full scan experiment (100-1500 m/z) at a nominal resolving power of 70,000 (referenced to m/z 200) and data-independent MS/MS experiments at a nominal resolving power of 35,000. For the latter, we acquired the data using broad isolation windows of about 50 mu (i.e., m/z ranges 97-147, 144-194, 191-241, 238-288, 285-335, 332-382, 379-429, 426-476) and 280 mu (i.e., m/z ranges 460-740, 730-1010), respectively.

The column oven was operated at 50°C and the flow rate was set to 0.3 mL/min. Equilibration time was 7 minutes.

Table S1-4: Solvent gradients for LC-HRMS analysis in positive and negative mode

Start (Min)	Final % solvent A	Final % solvent B
0	100	0
15	0	100
22	100	0
30	100	0

The internal standard mix consisted of the compounds listed in Table S1-3 and was at 1 µg/mL. Internal standards were assigned by nearest retention time or to the respective analyte without isotope label.

Table S1-5: Internal standard mix used for LC-HRMS

Mono-isobutylphthalate-D4	Decyltrimethylammonium-D30
Creatinine-D3	Atenolol-D7
Diazinon-D10	Progesterone-D9
Benzophenone-3-D5	Verapamil-D6
p-Toluene-sulfonamide-D4	Bezafibrate-D4
Cotinine-D3	Sulfamethoxazole-D4
Diglyme-D6	Tebuconazole-D9
Chlormequat-D9	Imidacloprid-D4
Carbamazepine-D10	4-Nitrophenol-D4
Atrazine-13C3	Triclosan-D3
Benzotriazole-D4	Mecoprop-D3
Carbendazim-D4	Acesulfame-D4
Tri-n-butylphosphate-D27	Hydrochlorothiazide-13C6
DEET-D7	Bentazone-D6
Metolachlor-D6	Cyclamate-D11
Isoproturon-D3	Clarithromycin-D3
Diclofenac-D4	Desisopropylatrazine-D5
Caffeine-D3	

Text S6. Time-dependence of recovery with PES

A total of 500 mg PDMS was prepared per sample by cutting 12 pieces of the size of 1 mm thickness.Total volume was 900 µL with the following sample types: A) 900 µL PBS ("blank"), B) 300 µL pooled plasma + 600 µL PBS ("unspiked plasma"), C) 64 µL of a 500 ng/mL spike mix which was blown down in a nitrogen stream, then adding 300 µL of pooled plasma and 600 µL PBS ("spiked plasma"). All samples were prepared in glass vials.

To investigate the necessary time for equilibration in human plasma, the extraction was performed with a chemical spike mixture with a lower number of components (identified by the respective column in Supporting Information S2, Table S2-1) in triplicates for one, three, and six days. After preparation, the samples were equilibrated on an orbital shaker with 400 rpm for the respective duration at 7.5°C. After this time, the PDMS was removed, washed in MilliQ water and tapped dry on lint-free tissue. The PDMS was extracted for one day with 3.5 mL of ethyl acetate on a horizontal shaker at room temperature. This step was repeated once. The extract was evaporated to dryness under nitrogen, transferred to 2 mL autosampler vials with 200 µL glass micro inserts and reconstituted in 40 µL of MeOH. The supernatant was prepared for SPE by adding 300 µL of 4% of formic acid. SPE was performed with 96-well SPE plates with HLB sorbent using a negative pressure unit with a suitable manifold. The SPE plate was conditioned with 1 mL of ethyl acetate, 1 mL of methanol, and 1 mL of water. The sample was transferred, extracted, and the sorbent washed with 0.5 mL of 5% MeOH in water. The plates were left under vacuum for 30 minutes and subsequently centrifuged at 1,500 × g for 30 minutes. The plates were left over night in a desiccator at room temperature to achieve full dryness. The plates were eluted using 1 mL of MeOH, which was collected in glass-coated 96-well plates. The eluate was evaporated to near dryness under nitrogen, transferred to 2 mL autosampler vials with 200 µL glass micro inserts, evaporated to dryness and reconstituted in 40 µL of MeOH.

As depicted in Figure S1-2, PDMS alone had a mean ± standard deviation recovery of 22 ± 23% (one day), 25 ± 25% (three days), and 25 ± 25% (six days) with n = 351. Details regarding respective recoveries can be accessed from Supporting Information S2, Table S2-2.

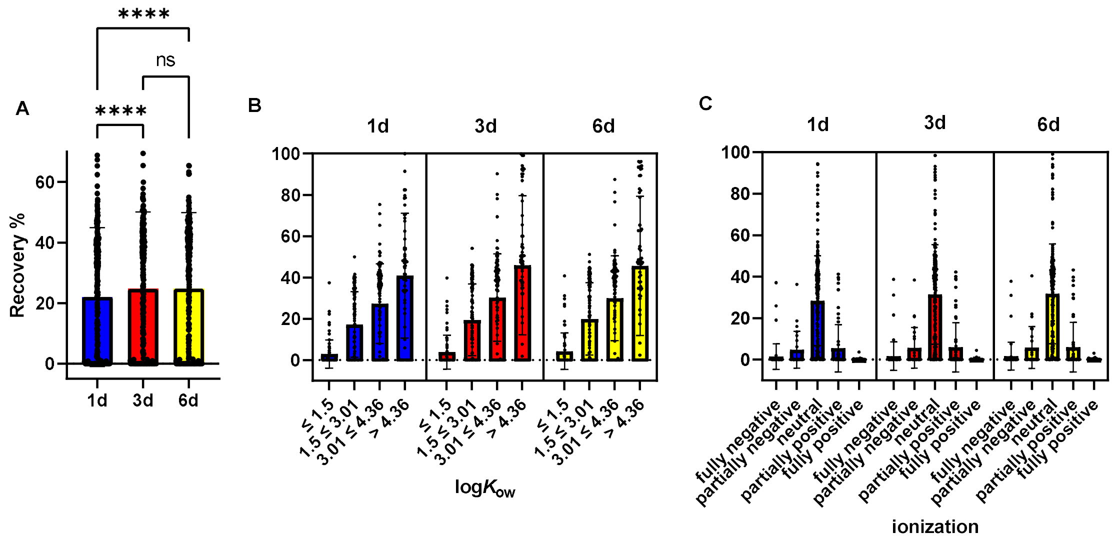

Figure S1-2: Chemical recoveries in % for PES at different timepoints.
(A) Overall mean recovery and standard deviation as well as individual for all analyzed compounds (n = 351). **(B)** Recoveries were divided in ranges of hydrophobicity: logK_{ow}: ≤ 1.5 (n = 70), 1.5 ≤ 3.01 (n = 106), 3.01 ≤ 4.36 (n = 108), > 4.36 (n = 67). **(C)** Recoveries binned according to ionization at pH 7.4: fully negative (n = 61), partially negative (n = 24), neutral (n = 254), partially positive (n = 62), fully positive (n = 32).1d = one day, 3d = three days, 6d = six days equilibration time. Plotted are all individual data points as black circles, the means as boxes as well as standard deviations of the mean as error bars. logK_{ow} = octanol-water partition constant. Significance tested by paired Friedman test and Dunn's multiple comparison; ns. not significant. Data in Table S2-2.

The rank means were significantly different with $p < 0.0001$ for paired Friedman test with recoveries after one day equilibration time being significantly lower ($p < 0.0001$) than three days and six days equilibration time. There was no significant difference in rank sums of recoveries after three days and six days equilibration ($p = 0.2791$).

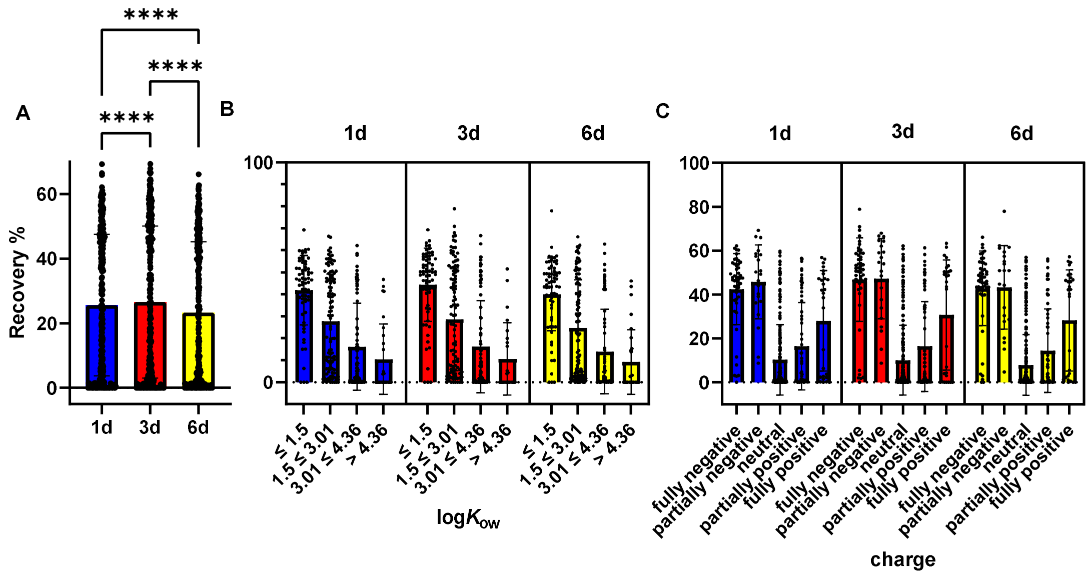

Figure S1-3: Chemical recoveries in % for SPE after PES for different durations (1, 3 or 6 days). **(A)** Overall mean recovery and standard deviation for all detected compounds (*n* = 285). **(B)** Recoveries were divided in ranges of hydrophobicity: logK_{ow}: ≤ 1.5 (*n* = 64), 1.5 ≤ 3.01 (*n* = 106), 3.01 ≤ 4.36 (*n* = 87), > 4.36 (*n* = 28). **(C)** Recoveries binned according to ionization at pH 7.4: fully negative (*n* = 49), partially negative (*n* = 21), neutral (*n* = 274), partially positive (*n* = 64), fully positive (*n* = 28). 1d = one day, 3d = three days, 6d = six days equilibration time. Plotted are all individual data points as black circles, the means as boxes as well as standard deviations of the mean as error bars. logK_{ow} = octanol-water partition constant. Significance tested by paired Friedman test and Dunn's multiple comparison. Data in Table S2-3.

For SPE after PDMS the mean ± standard deviation recoveries were 26 ± 22% (one day), 27 ± 24% (three days), and 23 ± 22% (six days) with *n* = 285. Individual chemical's recover can be accessed in the Supporting Information S2, Table S2-3. The rank means of all distributions were significantly different using paired Friedman test with p < 0.0001. All multiple comparisons were significantly different with p < 0.0001 with three days having the highest recoveries.

Text S7. PDMS-plasma partition constants

The $K_{PDMS/plasma}$ were converted from the recoveries with equation S(3) for all neutral chemicals (Table S2-7). The mean $K_{PDMS/plasma,i}$ per chemical *i* of chemicals with logK_{ow} > 2 did not show a strong dependence of hydrophobicity expressed as logK_{ow} (Figure S1-3, Table S2-7, mean per compound in Table S2-8). The slope of the linear regression over nine orders of magnitude was merely 0.12, and the $K_{PDMS/plasma}$ varied unsystematically over three orders of magnitude with a mean log$K_{PDMS/plasma}$ of -0.154 (standard deviation 0.80, 95% confidence interval -0.238 to -0.070, $K_{PDMS/plasma}$ 0.70) but there appears to be an upward trend at logK_{ow} ≤ 2. These $K_{PDMS/plasma}$ were approximately a factor of 10 to 100 lower than the $K_{PDMS/blood}$ measured in full blood of humans[9] and turtles,[9] despite the higher protein and lipid content of full blood.[10] The simple mass balance model (equation S(4)) provided a maximum estimate but overestimated the measured $K_{PDMS/plasma}$ (Figure S1-4) for many chemicals. The model and the data showed an apparent trend that partition constants were increasing from 0 < logK_{ow} < 2 and independent of hydrophobicity at logK_{ow} > 2.

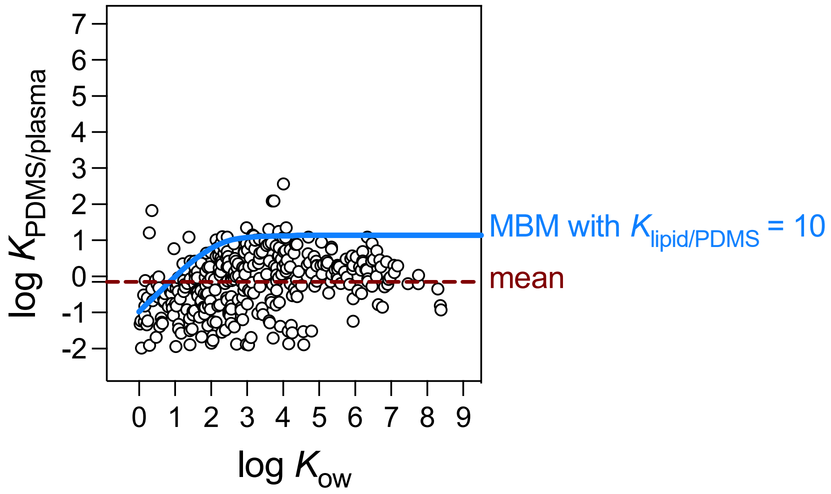

Figure S1-4: PDMS-plasma partition constants log$K_{PDMS/plasma}$ of the neutral chemicals (n = 352) as a function of the octanol-water partition constant logK_{ow}. Data from Table S2-8. red dashed line: mean of all log$K_{PDMS/plasma}$, blue line prediction by the mass-balance model (MBM) described by equation S(4) with $K_{lipid/PDMS}$ of 10.

Text S8. Calculation of standard errors for the prediction of mixture effects

For all directly fitted assay data, the standard errors were calculated using the included functions in GraphPad Prism assuming symmetric confidence intervals. For the mixture calculations, the error in the predicted effect concentration for effect level F of concentration addition, $EC_{CA,F}$, was calculated using equation S(5).

$$\sigma EC_{F,CA}=\sqrt{\sum_{i=1}^{n}\left(\left(\frac{EC_{F,CA}^{2}*p_{i}}{EC_{F,i}^{2}}\right)^{2}*\sigma EC_{i}^{2}\right)} \qquad S(5)$$

For independent action, first the errors of effects at level F per chemical *i* were calculated using equation S(6), which were then used to calculate the error of predicted mixture effects via equation S(7).

$$\sigma Effect_{F,i}=\sqrt{\left(\frac{\partial Effect_{F,i}}{\partial EC_{50,i}}\right)^{2}*\sigma EC_{50,i}^{2}+\left(\frac{\partial Effect_{F,i}}{\partial slope_{i}}\right)^{2}*\sigma slope_{i}^{2}} \qquad S(6)$$

$$\sigma Effect_{F,IA}=\sqrt{\sum_{i=1}^{n}\left(\left(\frac{\partial Effect_{F,mix}}{\partial Effect_{F,i}}\right)^{2}*\sigma Effect_{F,i}^{2}\right)} \qquad S(7)$$

The deviation of effect was calculated using equation S(8) at the effect level F of interest.

$$\sigma EC_{F,IA}=\sqrt{\left(\frac{\partial EC_{IA}}{\partial EC_{F,IA}}\right)^{2}*\sigma Effect_{F,IA}^{2}+\left(\frac{\partial EC_{IA}}{\partial EC_{50,IA}}\right)^{2}*\sigma EC_{50,IA}^{2}+\left(\frac{\partial EC_{IA}}{\partial slope_{IA}}\right)^{2}*\sigma slope_{IA}} \qquad S(8)$$

For AREc32 a linear evaluation was selected and hence the deviation was calculated according to equations S(9) and S(10).

$$\sigma slope_{mixture}=\sum_{i=1}^{n}p_{i}^{2}*\sigma slope_{i}^{2} \qquad S(9)$$

$$\sigma EC_{F,mixture}=\frac{F}{slope_{mixture}^{2}}*\sigma slope_{mixture} \qquad S(10)$$

Text S9. Index on Prediction Quality

The comparisons between predicted and measured mixture effects of the binary mixture of unspiked samples and reference chemical were expressed as index on prediction quality (IPQ, equation 7) and standard deviation (SD). IPQs are summarized for concentration addition in Table S1-6 and for independent action in Table S1-7.

Table S1-6: Index on prediction quality and standard deviation for concentration addition

			**PES+SPE**	**SPE**	**SolvPrec**
**Assay**	**Effect level F***	**Sample**	**IPQ±SD**	**IPQ±SD**	**IPQ±SD**
**SH-SY5Y**	50|50|10	Plasma1	2.27±0.72	1.31±0.55	-
	50|50|50	Plasma2	1.19±0.21	4.67±1.38	0.67±0.15
	50|50|50	Plasma3	0.65±0.17	2.80±1.80	0.29±0.13
		**Mean**	**1.36±26**	**2.93±0.78**	**0.48±0.10**
**AhR-CALUX**	20|20|50	Plasma1	0.59±0.12	0.68±0.20	0.40±0.17
	10|50|10	Plasma2	0.51±0.12	0.31±0.06	0.09±0.06
	10|50|10	Plasma3	0.11±0.05	0.48±0.14	-0.30±0.05
		**Mean**	**0.40±0.06**	**0.17±0.08**	**0.06±0.06**
**PPARγ-BLA**	10|10|30	Plasma1	1.18±0.37	3.68±1.62	1.83±0.38
	10|10|10	Plasma2	0.19±0.06	0.02±0.04	0.07±0.06
	30|30|30	Plasma3	0.10±0.10.	0.31±0.08	0.25±0.08
		**Mean**	**0.42±0.13**	**1.13±0.54**	**0.71±0.13**
**AREc32**	1.5|1.5|1.5	Plasma1	0.39±0.04	0.79±0.08	1.66±0.41
	1.5|1.5|1.5	Plasma2	0.60±0.08	3.71±0.73	1.57±0.14
	1.5|1.5|1.5	Plasma3	0.11±0.04	0.35±0.06	0.03±0.04
		**Mean**	**0.03±0.03**	**1.09±0.25**	**1.08±015**

Mean index on prediction quality (IPQ) ± standard deviation (SD) resampled with *n* = 95,000. *AREc32 Induction Ratio, else %.

Table S1-7: Index on prediction quality and standard deviation for independent action

			**PES+SPE**	**SPE**	**SolvPrec**
**Assay**	**Effect level F***	**Sample**	**IPQ±SD**	**IPQ±SD**	**IPQ±SD**
**SH-SY5Y**	50|50|10	Plasma1	1.33±0.5	0.59±0.36	-
	50|50|50	Plasma2	1.36±0.21	5.57±1.71	1.08±0.14
	50|50|50	Plasma3	0.33±0.09	0.64±0.73	0.09±0.01
		**Mean**	**1.00±018**	**2.27±0.63**	**0.58±0.07**
**AhR-CALUX**	20|20|50	Plasma1	0.79±0.21	0.63±0.22	0.97±0.18
	10|50|10	Plasma2	0.74±0.11	0.70±0.04	0.52±0.06
	10|50|10	Plasma3	0.35±0.03	0.10±0.09	0.14±0.02
		**Mean**	**0.62±0.08**	**0.41±0.08**	**0.54±0.06**
**PPARγ-BLA**	10|10|30	Plasma1	1.14±0.39	3.36±1.54	1.51±0.64
	10|10|10	Plasma2	0.24±0.06	0.18±0.04	0.24±0.05
	30|30|30	Plasma3	-	0.44±0.07	0.12±0.05
		**Mean**	0.35±0.11<br>**0.34±0.14**	**1.03±0.51**	**0.62±0.21**
**AREc32**	1.5|1.5|1.5		IA not applicable for AREc32[11]		

Mean index on prediction quality (IPQ) ± standard deviation (SD) resampled with *n* = 95,000. *AREc32 Induction Ratio, else %.

Text S10. Effect recoveries

The effect recoveries (ER) ± standard deviation (SD) per bioassay and endpoint are summarized in Table S1-8.

Table S1-8: Effect recoveries (ER) per method and bioassay/cell line calculated with equation (3) from the effect concentrations in Table S2-6.

				PES+SPE	SPE	SolvPrec
**Assay**	**Effect level F****	**Endpoint**	**Sample**	**ER±SD %**	**ER±SD %**	**ER±SD %**
**SH-SY5Y**	30|30|30	Neurite outgrowth	Plasma1	143±95	0±0	260±38
	30|30|30		Plasma2	67±43	17±10	420±100
	30|30|30		Plasma3	308±79	71±46	0±0
			**Mean**	**173±44**	**30±16**	**227±36**
**AhR-CALUX**	10|10|10	Cytotoxicity	Plasma1	259±0	21±3	243±38
	30|30|30		Plasma2	47±9	45±7	124±17
	50|50|50		Plasma3	33±6	33±5	117±33
			**Mean**	**113±4**	**33±4**	**162±18**
**AhR-CALUX**	30|30|30	AhR activation*	Plasma1	1195±0	12±2	1274±0
	20|30|10		Plasma2	783±0	30±4	463±0
	20|30|20		Plasma3	16±3	0±0	17±4
			**Mean**	**665±1**	**14±2**	**585±2**
**PPARγ-BLA**	50|50|50	Cytotoxicity	Plasma1	106±13	94±11	162±32
	50|50|50		Plasma2	67±10	81±12	74±18
	50|30|50		Plasma3	293±35	327±78	213±49
			**Mean**	**156±13**	**168±27**	**150±21**
**AREc32**	10|10|10	Cytotoxicity*	Plasma1	-	0±0	25±4
	10|10|10		Plasma2	0±0	31±4	41±6
	10|10|10		Plasma3	33±9	59±8	142±18
			**Mean**	**15±4**	**30±3**	**70±7**
**AREc32**	1.5|1.5|1.5	Nrf2 activation	Plasma1	148±21	40±7	182±26
	1.5|1.5|1.5		Plasma2	72±13	27±3	-
	1.5|1.5|1.5		Plasma3	88±11	40±6	-
			**Mean**	**103±10**	**36±4**	**182±26**

Mean effect recovery (ER) ± standard deviation (SD) resampled with n = 95,000. *poor fit of concentration response data. **AREc32 Induction Ratio, else %.

Text S11. Concentration-response curves of bioassays

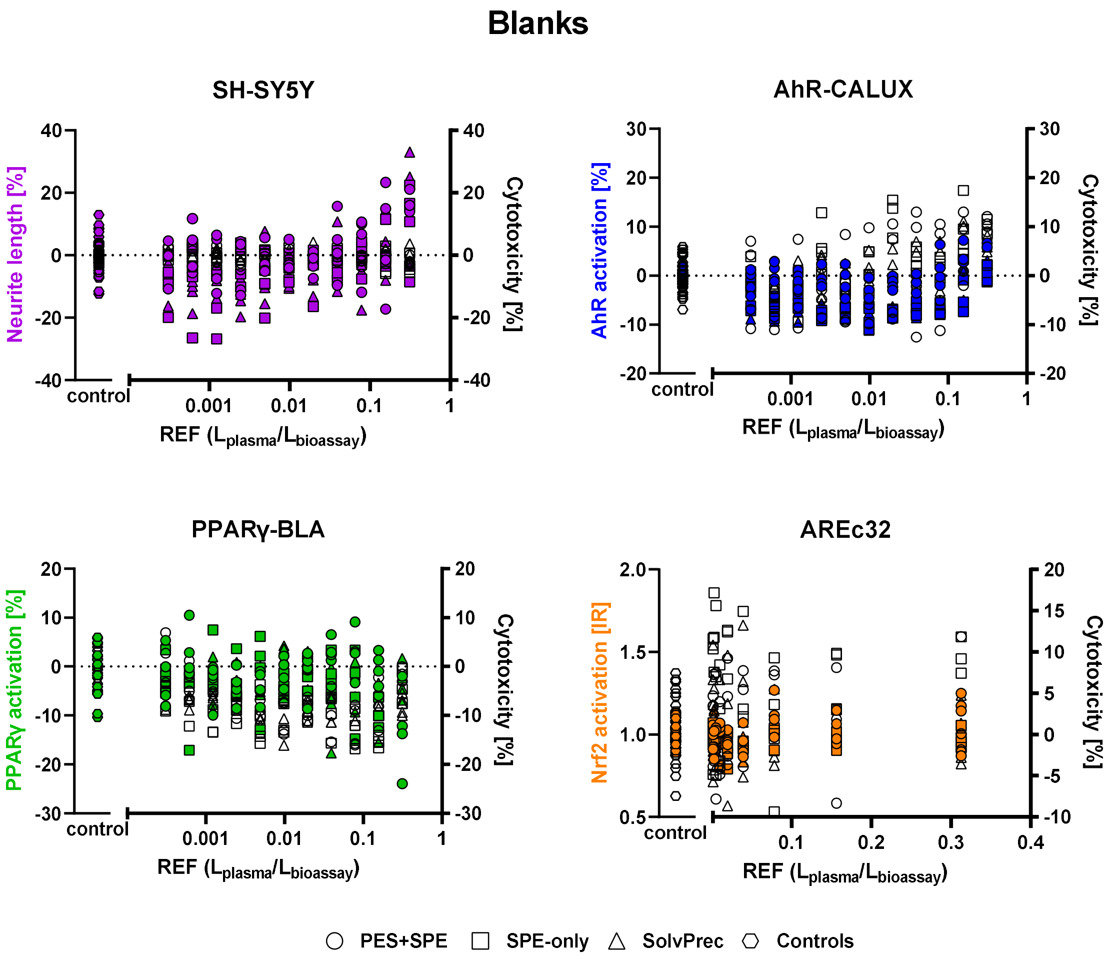

Figure S1-5: Concentration-response curves of method blanks in all cell lines.
Effect and inhibitory concentration values (EC_F and IC_F) listed in Table S2-6.

References

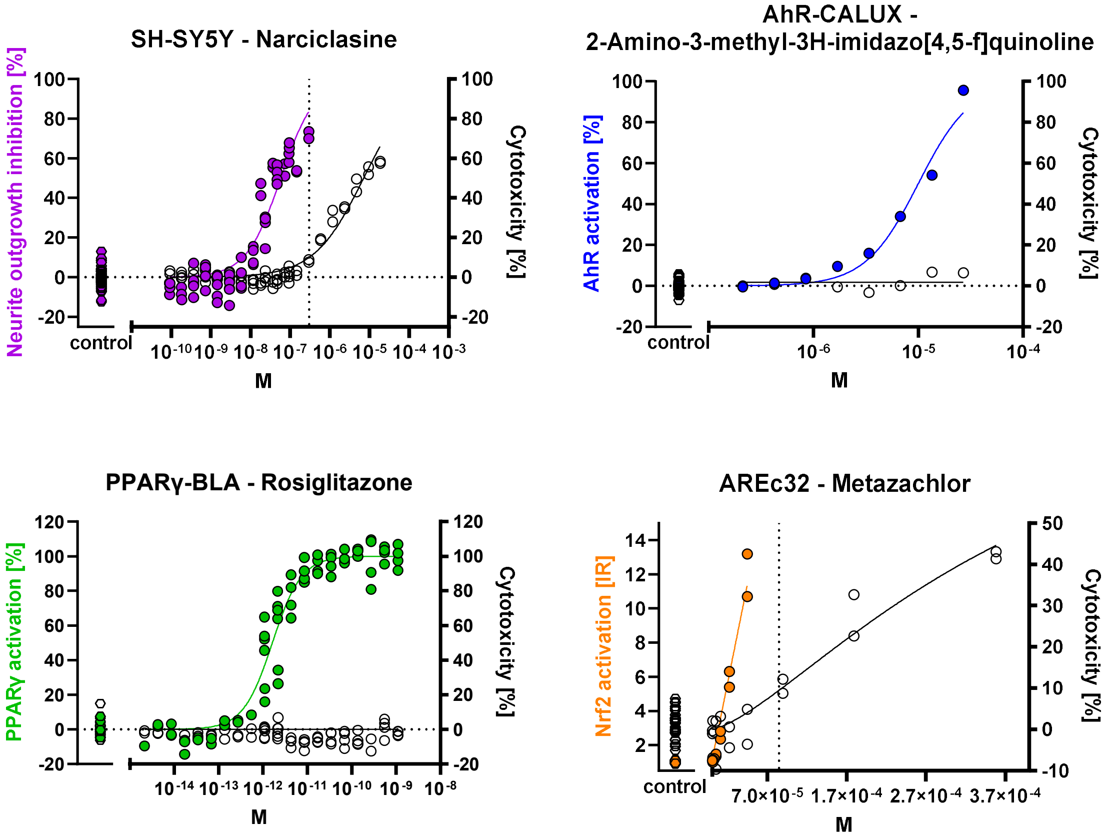

Figure S1-6: Concentration-response curves of single reference chemicals used for the binary mixture experiments.
The vertical dotted line indicates the inhibitory concentration at 10% cytotoxicity (IC_{10}). Effect and inhibitory concentration values (EC_F or $EC_{IR1.5}$ for AREc32 and IC_{10}) are listed in Table S2-6.

Spike mix

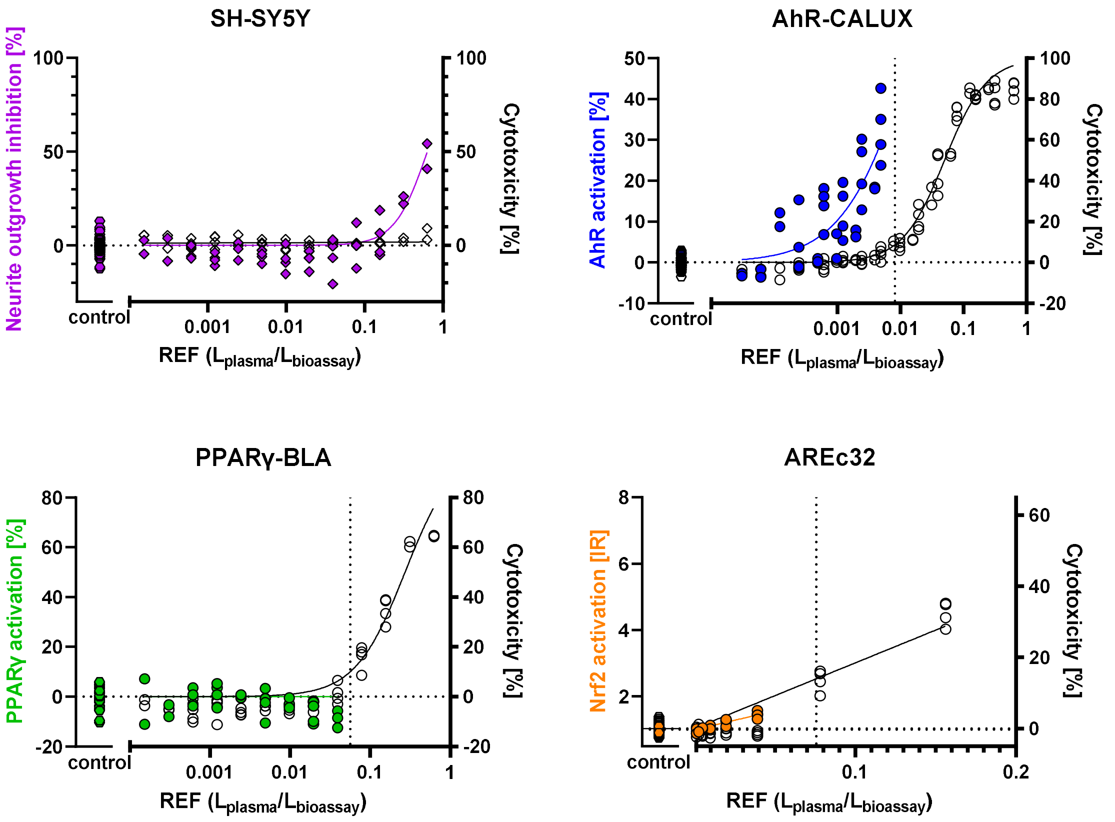

Figure S1-7: Concentration-response curves of the spike mix in all cell lines.
The vertical dotted line indicates the inhibitory concentration at 10% cytotoxicity IC_{10}). Effect and inhibitory concentration values (EC_F or $EC_{IR1.5}$ for AREc32 and IC_{10}) are listed in Table S2-6.

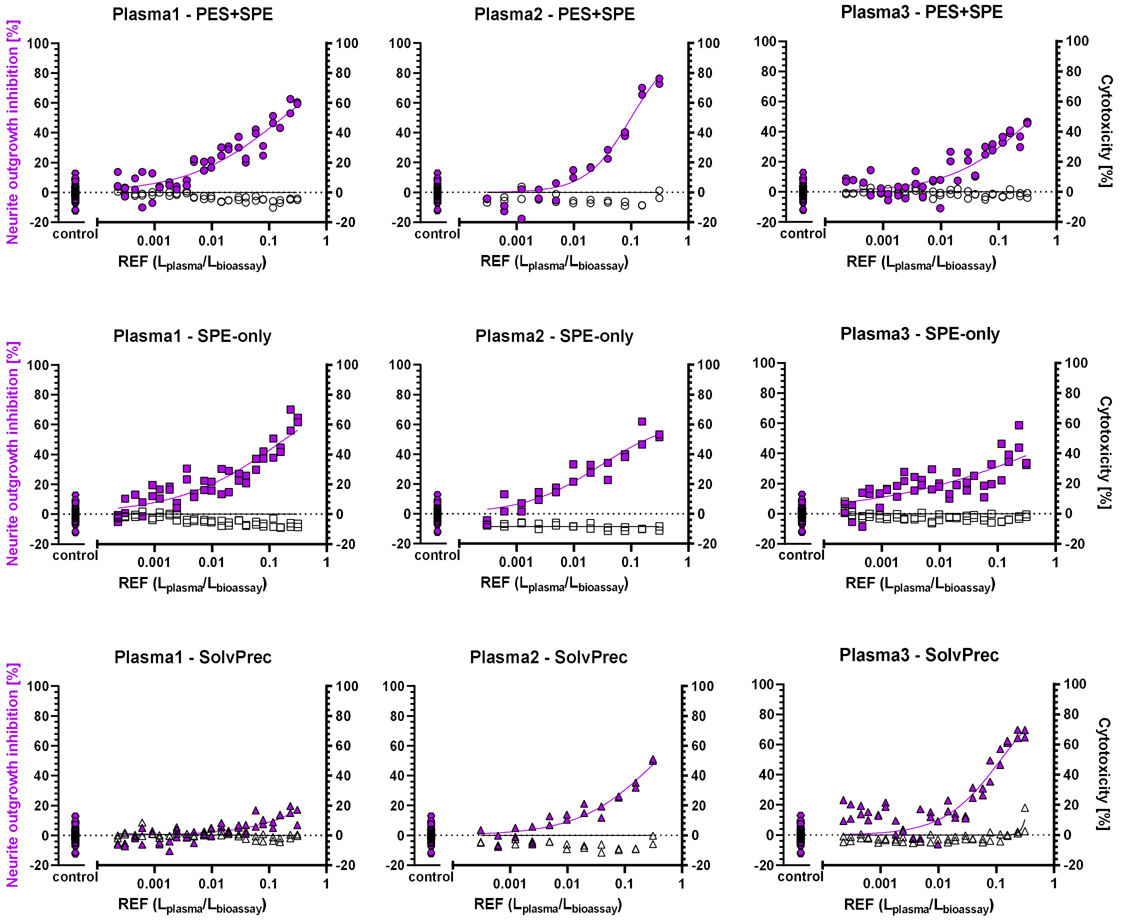

Figure S1-8: Concentration-response curves of unspiked plasma samples in SH-SY5Y. IC$_{10}$). Effect and inhibitory concentration values (EC$_F$ and IC$_{10}$) are listed in Table S2-6.

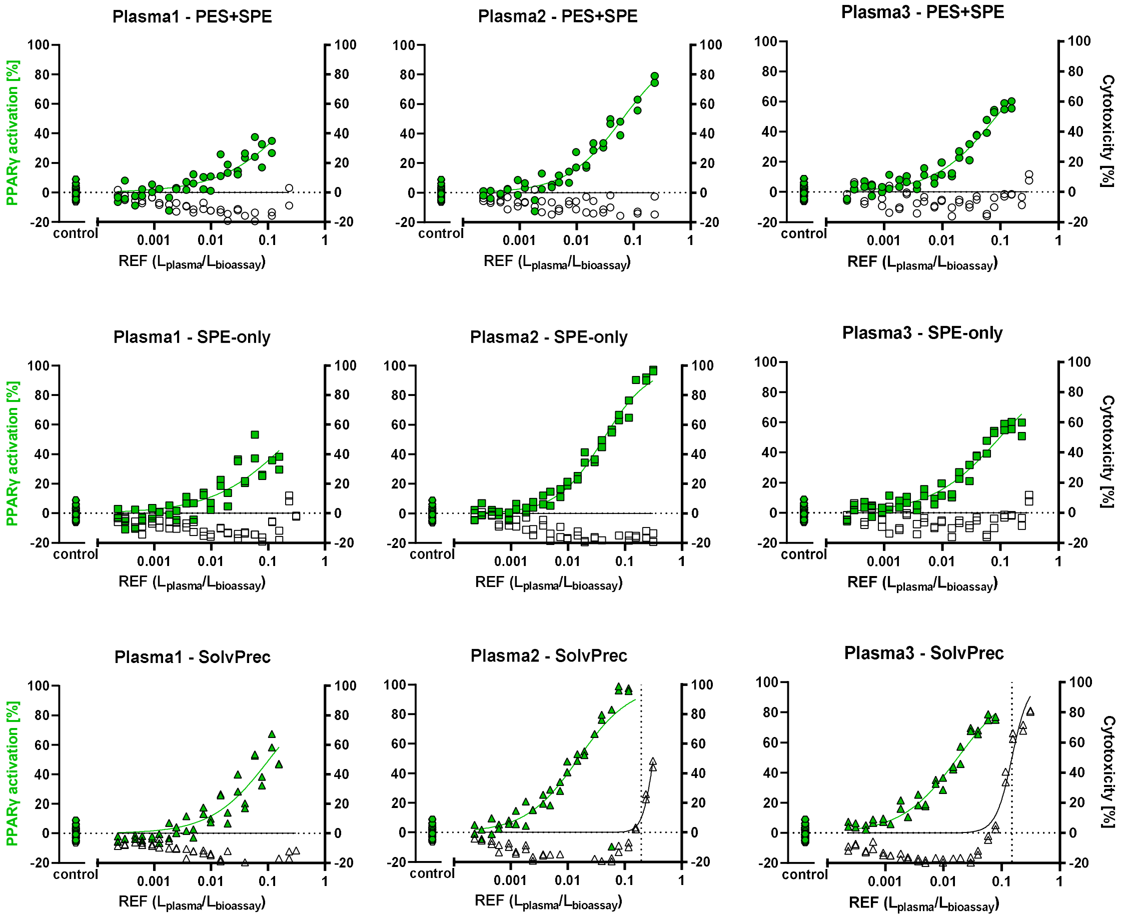

Figure S1-9: Concentration-response curves of unspiked plasma samples in PPARγ-BLA. The vertical dotted line indicates the inhibitory concentrations at 10% cytotoxicity Effect and inhibitory concentration values (EC_F and IC_{10}) are listed in Table S2-6.

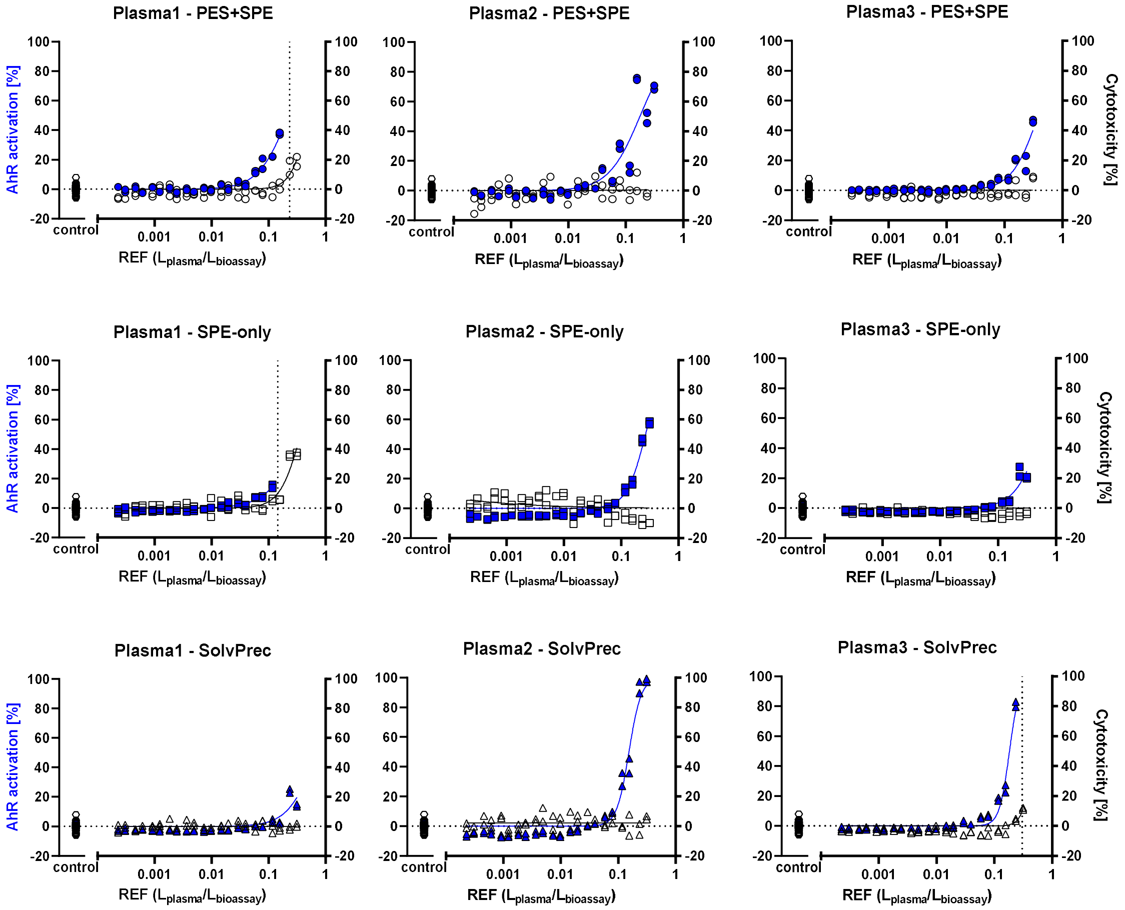

Figure S1-10: Concentration-response curves of unspiked plasma samples in AhR-CALUX. The vertical dotted line indicates the inhibitory concentration at 10% cytotoxicity Effect and inhibitory concentration values (EC_F and IC_{10}) are listed in Table S2-6.

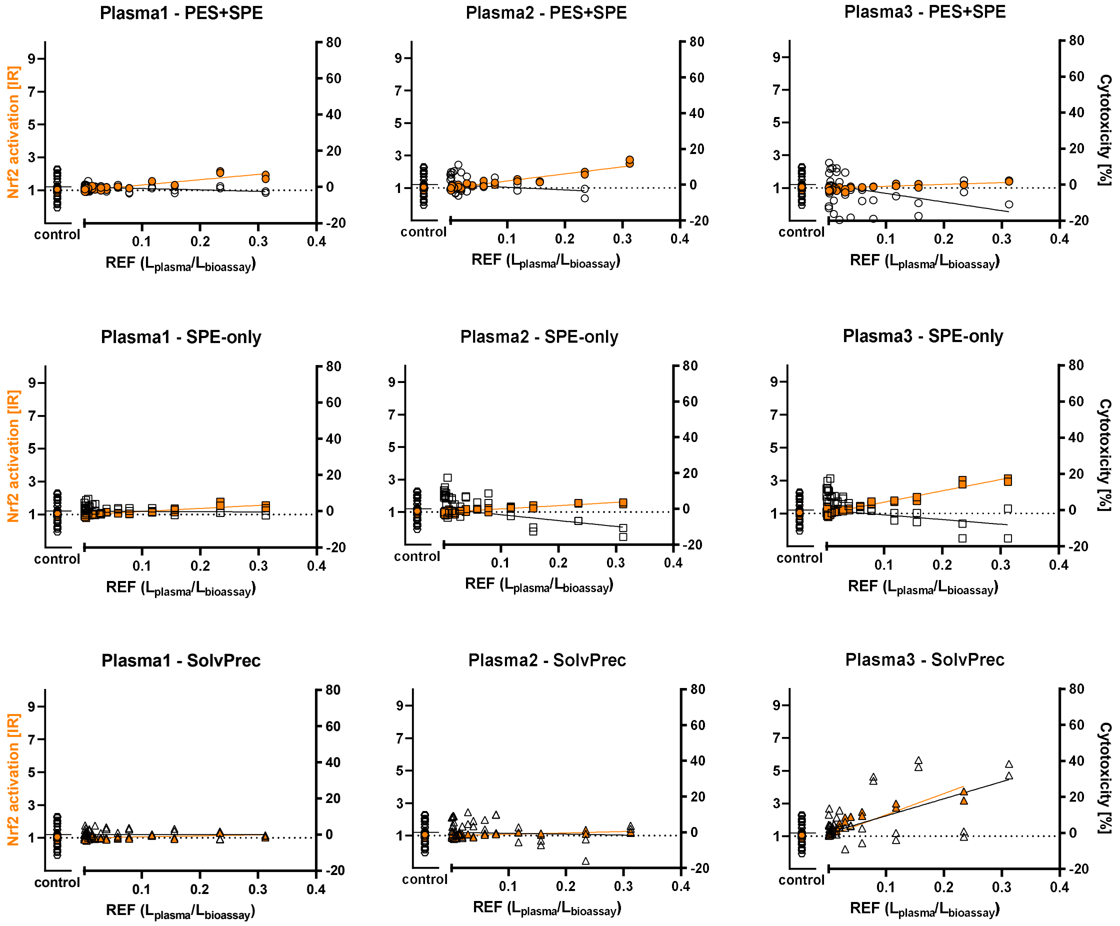

Figure S1-11: Concentration-response curves of unspiked plasma samples in AREc32. Effect and inhibitory concentration values (EC_F and IC_{10}) are listed in Table S2-6.

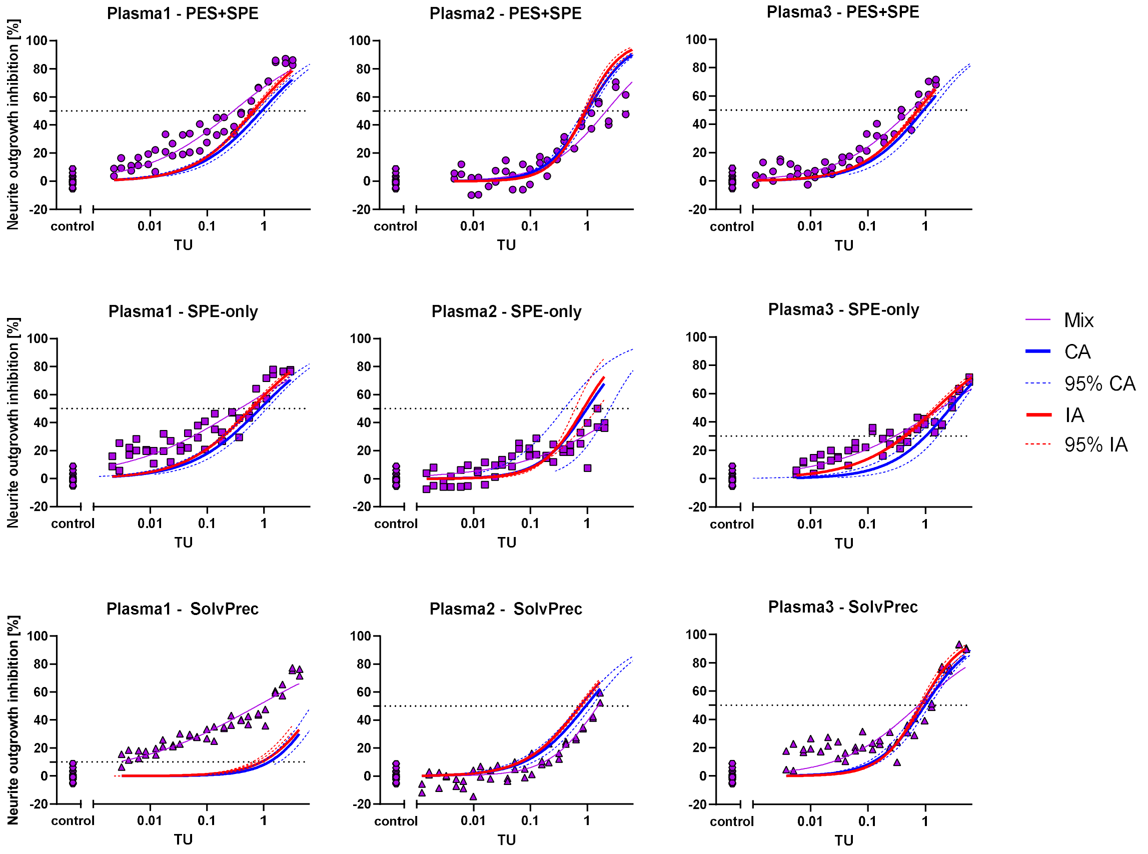

Figure S1-12: Concentration-response curves of unspiked plasma + reference in SH-SY5Y. Purple = measured response of unspiked plasma + Narciclasine, blue = prediction by concentration addition (CA), red = prediction by independent action (IA). Dotted lines = 95% interval as ± 1.96 * standard error. Horizontal dotted line = effect level F. Effect and inhibitory concentrations (EC_F and IC_{10}) are listed in Table S2-6.

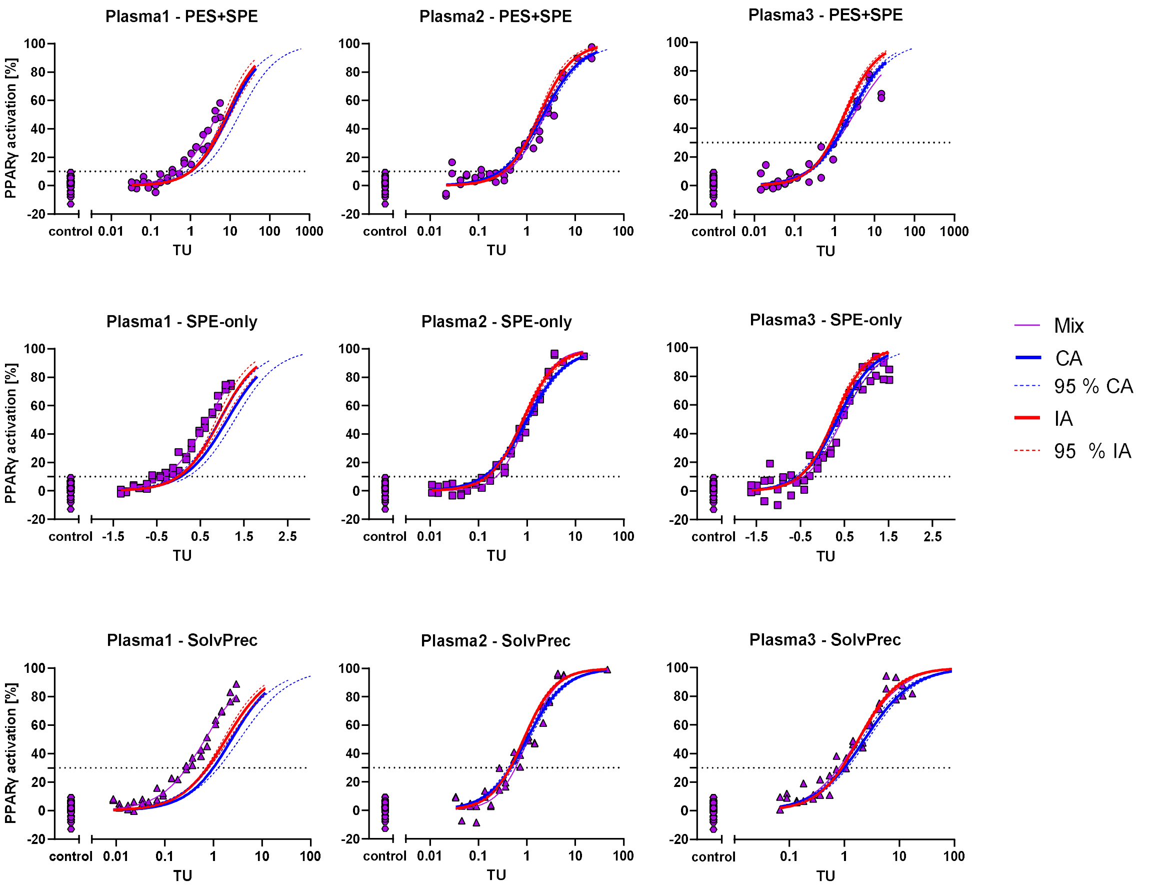

Figure S1-13: Concentration-response curves of unspiked plasma + reference in PPARγ-BLA. Purple = measured response of unspiked plasma + Rosiglitazone, blue = prediction by concentration addition (CA), red = prediction by independent action (IA). Dotted lines = 95% interval as ± 1.96 * standard error. Horizontal dotted line = effect level F. Effect and inhibitory concentrations (EC_F and IC_{10}) are listed in Table S2-6.

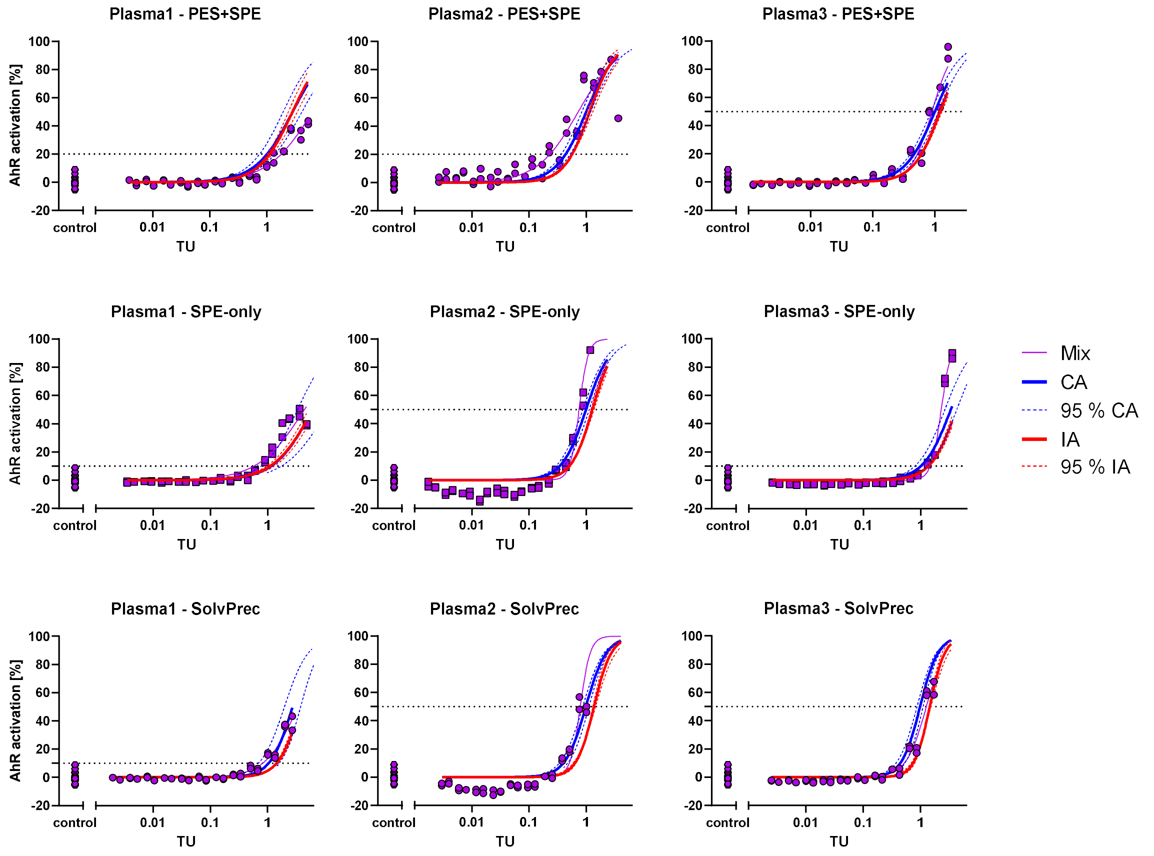

Figure S1-14: Concentration-response of unspiked plasma + reference in AhR-CALUX. Purple = measured response of unspiked plasma + 2-Amino-3-methyl-3H-imidazo[4,5-f]quinoline, blue = prediction by concentration addition (CA), red = prediction by independent action (IA). Dotted lines = 95% interval as ± 1.96 * standard error. Horizontal dotted line = effect level F. Effect and inhibitory concentrations (EC_F and IC_{10}) are listed in Table S2-6.

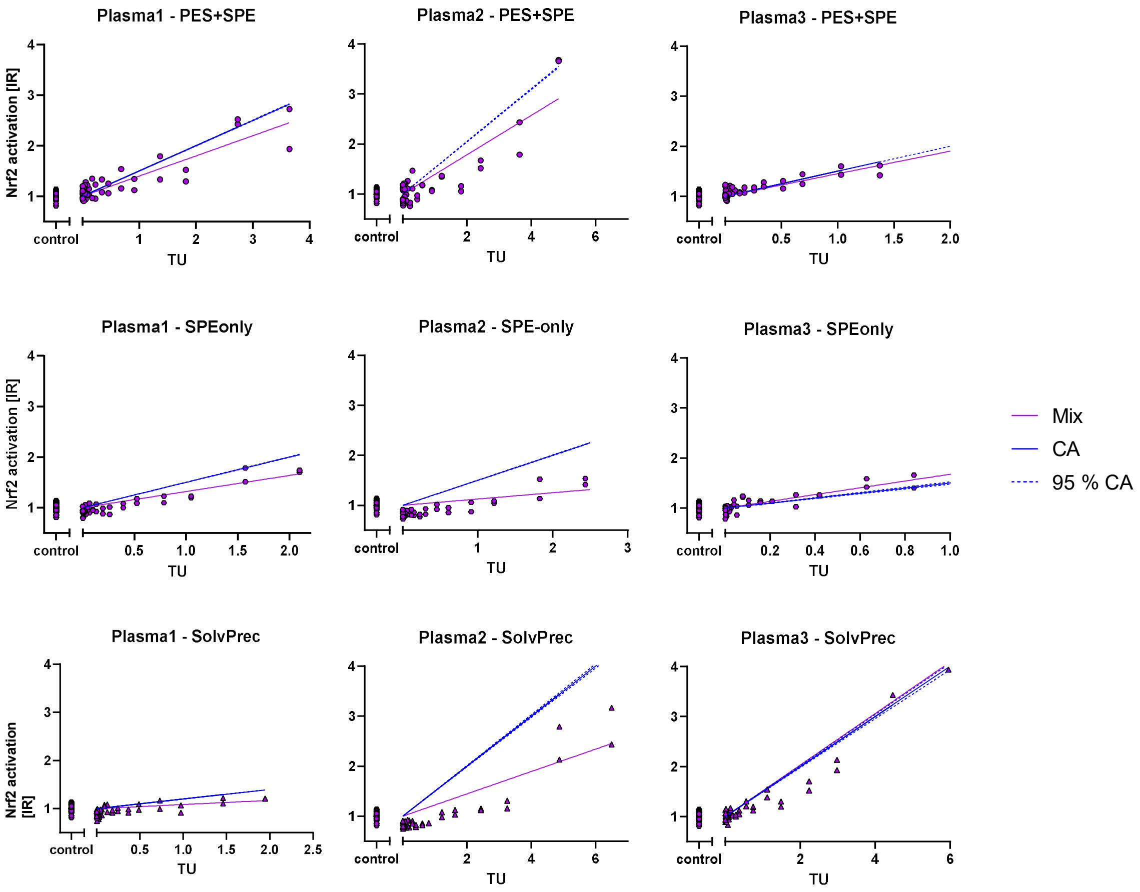

Figure S1-15: Concentration-response of unspiked plasma + reference in AREc32.
Purple = measured response of unspiked plasma + Metazachlor, blue = prediction by concentration addition (CA). Dotted lines = 95% interval as ± 1.96 * standard error. Horizontal dotted line = effect level F. Effect and inhibitory concentrations ($EC_{IR1.5}$ and IC_{10}) are listed in Table S2-6.

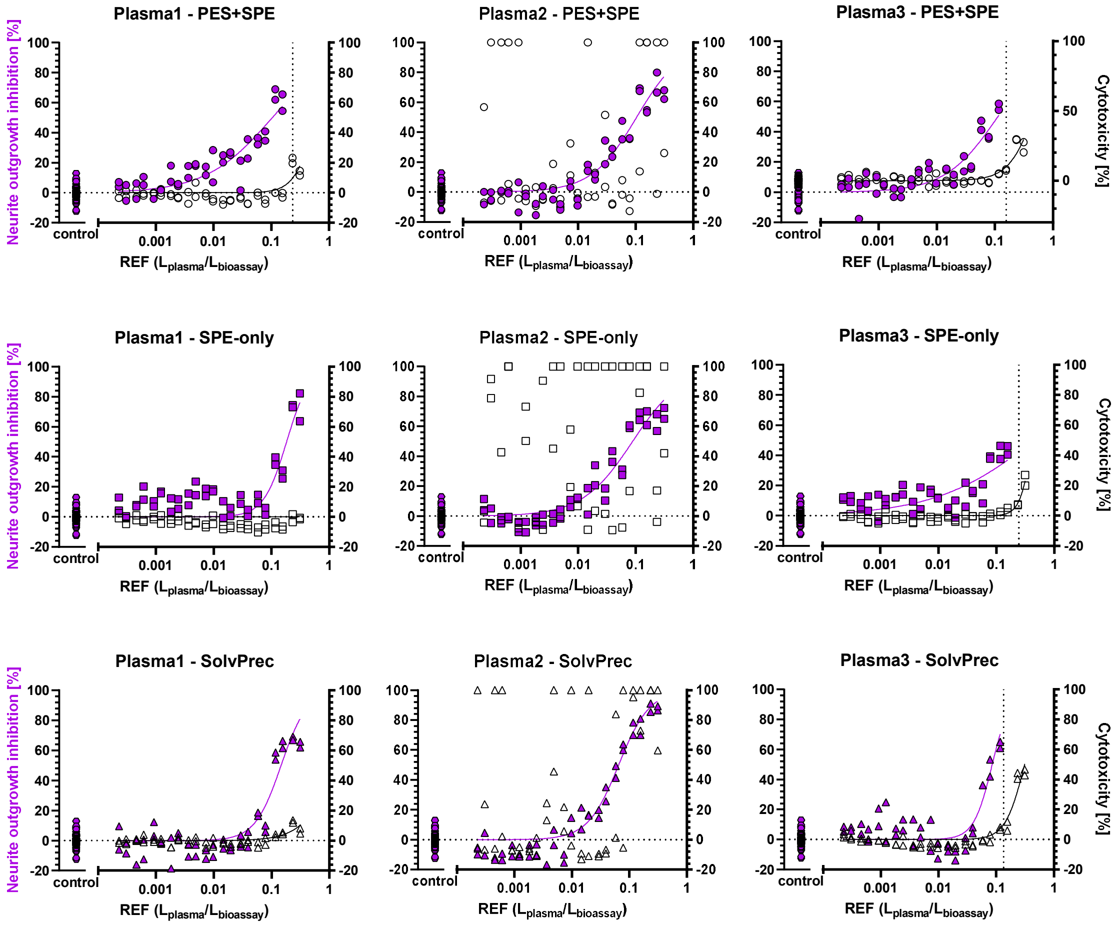

Figure S1-16: Concentration-response curves of spiked plasma samples in SH-SY5Y. A liquid handling error in Plasma2 resulted in a staining error. The respective cytotoxicity data was not used in any analysis. The vertical dotted line indicates the inhibitory concentration at 10% cytotoxicity. Effect and inhibitory concentrations (EC_F and IC_{10}) are listed in Table S2-6.

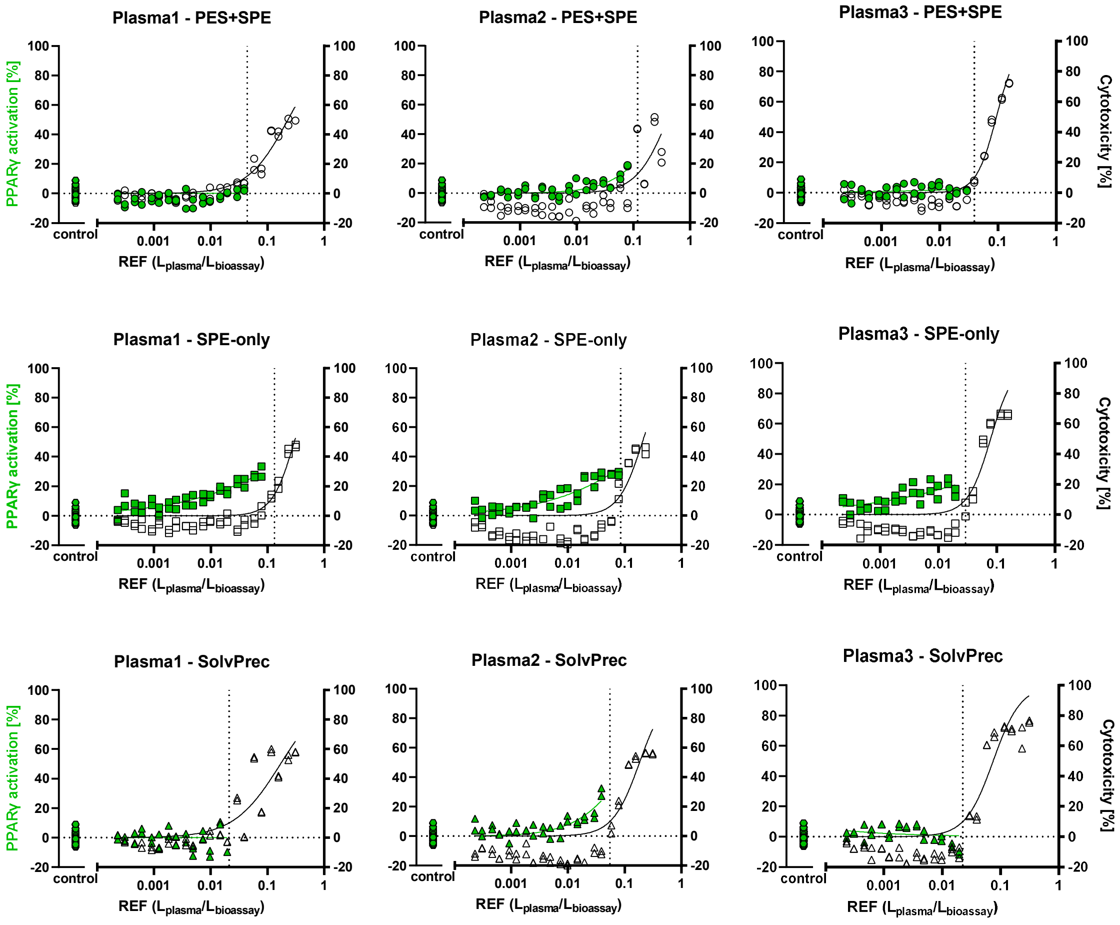

Figure S1-17: Concentration-response curves of spiked plasma samples in PPARγ-BLA.
The vertical dotted line indicates the inhibitory concentration at 10% cytotoxicity. Effect and inhibitory concentration values (EC_F and IC_{10}) listed in Table S2-6.

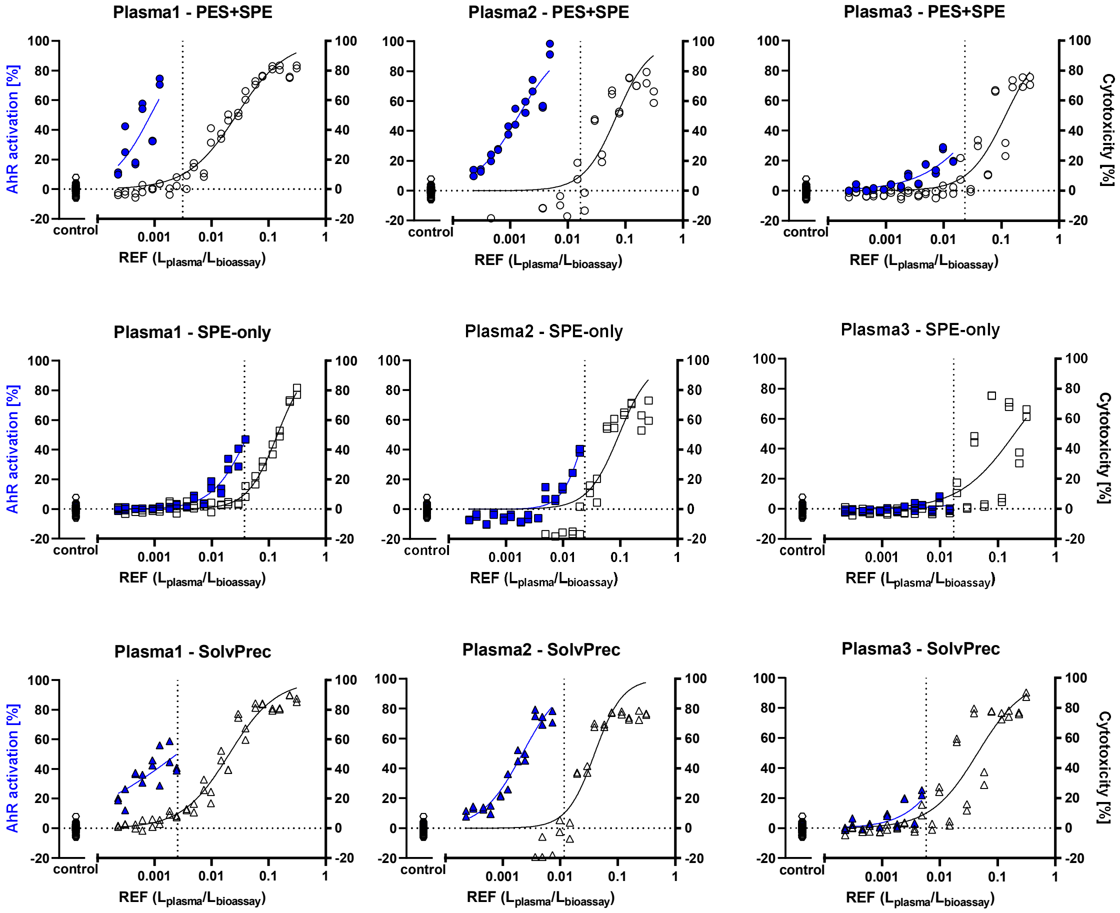

Figure S1-18: Concentration-response curves of spiked plasma samples in AhR-CALUX.
The vertical dotted line indicates the inhibitory concentration at 10% cytotoxicity. Effect and inhibitory concentration values (EC_F and IC_{10}) listed in Table S2-6.

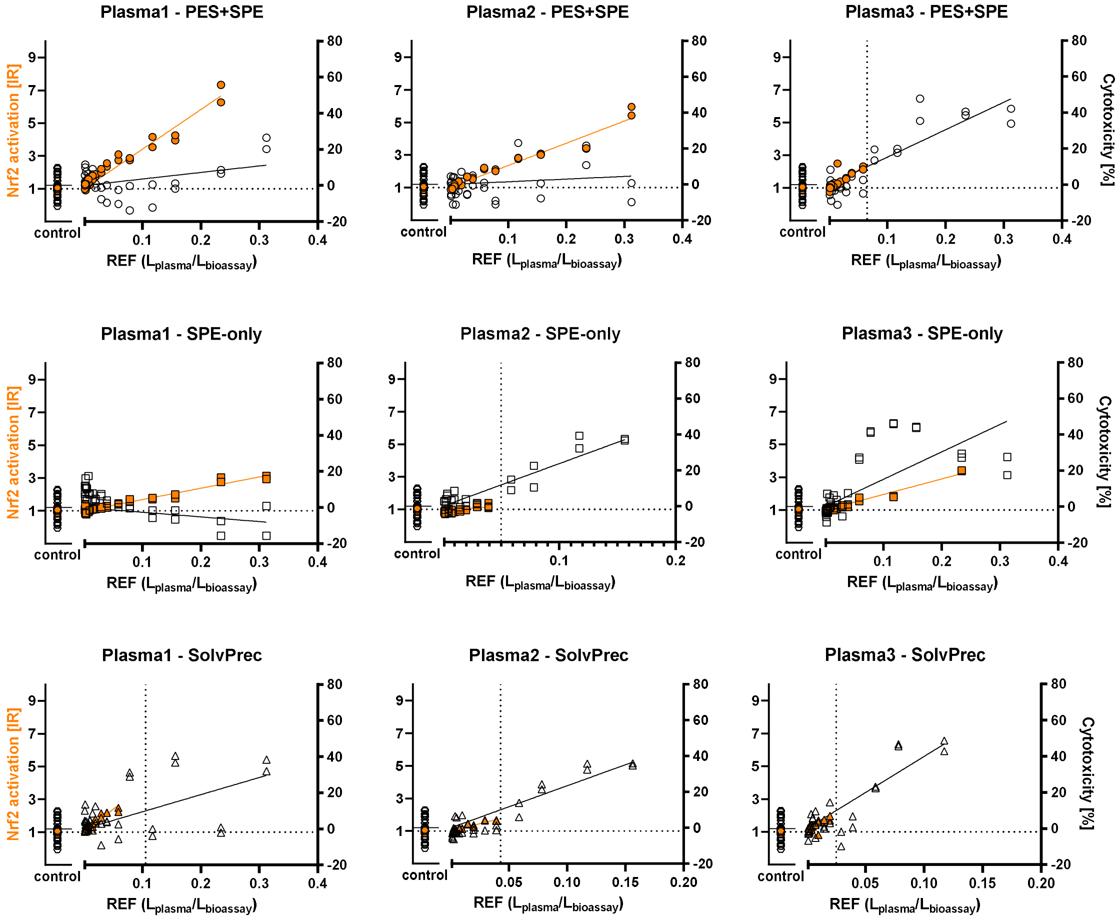

Figure S1-19: Concentration-response curves of spiked plasma samples in AREc32.
The vertical dotted line indicates the inhibitory concentration at 10% cytotoxicity. Effect and inhibitory concentration values (EC_F and IC_{10}) listed in Table S2-6.

References

1. Lee, J.; Braun, G.; Henneberger, L.; König, M.; Schlichting, R.; Scholz, S.; Escher, B. I., Critical membrane concentration and mass-balance model to identify baseline cytotoxicity of hydrophobic and ionizable organic chemicals in mammalian cell lines. *Chemical Research in Toxicology* **2021,** *34* (9), 2100-2109.
2. Reiter, E. B.; Jahnke, A.; König, M.; Siebert, U.; Escher, B. I., Influence of co-dosed lipids from biota extracts on the availability of chemicals in in vitro cell-based bioassays. *Environmental Science & Technology* **2020,** *54* (7), 4240-4247.
3. Ismaiel, O. A.; Zhang, T.; Jenkins, R. G.; Karnes, H. T., Investigation of endogenous blood plasma phospholipids, cholesterol and glycerides that contribute to matrix effects in bioanalysis by liquid chromatography/mass spectrometry. *Journal of Chromatography B* **2010,** *878* (31), 3303-3316.
4. Little, J. L.; Wempe, M. F.; Buchanan, C. M., Liquid chromatography–mass spectrometry/mass spectrometry method development for drug metabolism studies: examining lipid matrix ionization effects in plasma. *Journal of Chromatography B* **2006,** *833* (2), 219-230.
5. Carmical, J.; Brown, S., The impact of phospholipids and phospholipid removal on bioanalytical method performance. *Biomedical Chromatography* **2016,** *30* (5), 710-720.
6. Niu, L.; Henneberger, L.; Huchthausen, J.; Krauss, M.; Ogefere, A.; Escher, B. I., pH-dependent partitioning of ionizable organic chemicals between the silicone polymer polydimethylsiloxane (PDMS) and water. *ACS Environmental AU* **2022,** *2* (3), 253-262.
7. Baumer, A.; Jäsch, S.; Ulrich, N.; Bechmann, I.; Landmann, J.; Stöver, A.; Escher, B. I., Chemical mixtures in human post-mortem tissues assessed by a combination of chemical analysis and in vitro bioassays after extraction with silicone. *Environment International* **2021,** *157*, 106867.
8. Jin, L.; Gaus, C.; van Mourik, L.; Escher, B. I., Applicability of passive sampling to bioanalytical screening of bioaccumulative chemicals in marine wildlife. *Environmental Science & Technology* **2013,** *47* (14), 7982-7988.
9. Baumer, A.; Jäsch, S.; Ulrich, N.; Bechmann, I.; Landmann, J.; Escher, B. I., Kinetics of Equilibrium Passive Sampling of Organic Chemicals with Polymers in Diverse Mammalian Tissues. *Environmental Science & Technology* **2021,** *55* (13), 9097-9108.
10. Endo, S.; Brown, T. N.; Goss, K.-U., General model for estimating partition coefficients to organisms and their tissues using the biological compositions and polyparameter linear free energy relationships. *Environmental Science & Technology* **2013,** *47* (12), 6630-6639.
11. Escher, B. I.; van Daele, C.; Dutt, M.; Tang, J. Y.; Altenburger, R., Most oxidative stress response in water samples comes from unknown chemicals: the need for effect-based water quality trigger values. *Environmental Science & Technology* **2013,** *47* (13), 7002-7011.
